# Historical Sketch of the Progress of Medical Science, from June 1818, to January 1819

**Published:** 1819-01

**Authors:** 


THE LONDON

### Medical and Physical Journal.

1 OF VOL. XLI.] JANUARY, 1819. [NO. 239.

" For many fortunate discoveries in medicine, and for the detection of nume-
" rous errors, the world is indebted to the rapid circulation of Monthly
" Journals; and there never existed any work to which the Faculty in
" EUROPE and AMERICA were under deeper obligations than to the
" Medical and Physical Journal of London, now forming a long, but an
" invaluable, series."—RUSH.

### *Historical Sketch of the Progress of Medical Science, from June* 1818, *to January* 1819.

" Δεῖ γε μὴν, ταῦτα εἰδότα, μὴ λογισμῷ πρότερον πιθανῷ προσέχοντα ἰητρεύειν, ἀλλὰ τριβῇ μετὰ λόγου.—Ξυγκαταινέω μὲν οὖν και τὸν λογισμὸν, ἤν περ εκ περιπτώσιος ποιῆται τὴν ἀρχὴν, καὶ τὴν καταφορην ἐκ τῶν φαινομένων μεθοδεύη.—ει δὲ μὴ ἐξ ἐναργέος ἐφόδου, ἐκ δὲ πιθανῆς ανὰ πλάσιος λόγου, πολλάκις βαρέιην καὶ ἀνιαρην ἐπήνεγκε διάθεσιν."

ἹΠΠΟΚΡΑΤΗΣ.

IN tracing the following Sketch of the Progress of Medical Science, we propose to ourselves, as an example, the conduct of the reflective moral historian; who, after having taken a general view of the past, abstracts from the common mass those events which appear to be the centres of influence to others; and such as, although now apparently isolated and inefficient, seem to be calculated to produce important effects at a future period; relating them without strict regard to chronological order, or being detained in his course by common accidents or ephemeral objects, the neglect of which will not leave a blank in the history of the progress of the human mind.

We perceive that, to treat the history of medicine in this manner, is an arduous, and somewhat rash, undertaking; since no correct judgment can be formed of the value of facts that relate to it, beyond that which may be acquired from reflection on the effects we actually witness from their application. Many, that appear trifling to the historian, may be the sources whence the most important inferences may be derived. This is shewn in a forcible manner by the history of some other branches of science, the principles of which, instead of being merely suppositious, like those of medicine, are evident and intelligible; if there be any degree of certainty in the conclusions of the human understanding: thus, the laws which perpetuate the motions of the planetary system were ascertained from reasoning on the cause that makes an apple fall from a tree.

Those reflections, however, although they may add to our caution, and excite us to increased exertion, will not induce us to alter the plan of our undertaking. For, to give a particular relation of all the observations which have been detailed during the period comprised in this history, would occupy the space of large volumes; and merely to notice them in a general manner, would be worse than useless; since it might lead some persons to imagine that they had thus acquired information of all that was interesting, whilst, in reality, but very little real knowledge would be thus conveyed. In the prosecution of our object, we shall correct, to a certain degree, the errors into which we may now fall, by relating in subsequent annals, when the further development of their application shall convince us of their importance, the useful facts which at present are passed over without notice.

We may, perhaps, be permitted to say a few words more in favour of the conduct we have chosen to adopt. It will enable us to comprise, in the limits within which this history must necessarily be confined, a useful and sufficient account of all that, to the best of our judgment, has occurred in the progress of medical science really conducive to its improvement. It will also permit us to insert, occasionally, such illustrations of the facts we have to relate, as sources of information accessible to but few of our readers, and assiduous attention to this subject may enable us to adduce. A detail of facts is, however, our chief end; and, in the choice of them, we profess a perfect freedom from undue influence from the laws of any system, or the arguments of any sect. A philosopher, the correctness of whose opinions on the powers of the human understanding cannot be doubted, has impressed on our minds a sentiment which we hope will ever influence our conduct:—"Il est bien aisé sur des fondemens avouéz, de bastir ce qu'on veut; car selon la loy et ordonnance de ce commencement, le reste des pieces du bastiment se conduit aisément, sans se démentir. Par cette voye, nous trouvons nostre raison bien fondée, et discourons à bouleveüe."*

The epigraph we have chosen indicates the course we shall pursue: and this will necessarily lead us to devote a particular degree of attention to the observations of the physicians of that nation which must be admitted to be the centre whence knowledge in physical science is at present

* Montaigne, *Essais*, l. ii. c. xii.

most extensively diffused,—who appear to us most closely to follow the footsteps, and be guided by the advice, of him whom we consider took the only course by which true medical knowledge can be acquired. It is those physicians who, by the zeal with which they have cultivated *clinical medicine*, have most contributed to destroy that disposition to be the founders of new systems, which formerly constituted the characteristic of such as desired to acquire an eminence above their contemporaries. This is no longer considered as the only means to acquire fame and honour, and of fulfilling with propriety the serious duties of the professor of the healing art.

We cannot, therefore, refrain from expressing the pleasure we feel on observing that such principles for the study of medicine as those inculcated in the *Nosographie Philosophique* are now generally adopted throughout the greater part of Europe: those principles have directed the conduct of the most enlightened physicians of the present age, and have led them to contemn those doctrines, and artificial arrangements of diseases, which, not being founded on or confirmed by experience, are only to be considered as specimens of the aberrations of the understanding from the path which, if pursued, would have conducted it to that knowledge, beneficial to human nature.

It is in consequence of this disposition to observe facts, rather than frame hypotheses, that we have nothing to relate respecting imaginary splendid discoveries, or which is calculated to lead the enthusiast to suppose that the existing period is to be considered as an æra from which future acquirements in medical science will be dated; or of new systems, that, in the opinion of their infatuated authors, will, by the blaze of truth with which they presumptuously imagine them to be enlightened, show that those principles which have hitherto obtained attention and confidence were dictated by ignorance and delusion. We are, however, enabled to relate what will prove that genius and industry have not been engaged in the cultivation of medical science, without making acquirements that tend to substantiate opinions previously considered as doubtful, and which appear also to render intelligible what we had hitherto contemplated in vain.

It must not, however, be supposed that, because we have expressed disinclination to be guided by hypotheses, that we disallow their utility, or would have them banished from medical arguments. Theories, now apparently confirmed by long observation and experience, have in the course of their development been hypotheses; and absolutely to exclude these, would be to confine the operation of reason to such limits as would almost entirely arrest the progress of knowledge. We would merely desire that hypotheses should not usurp that place in our reasonings which should be conceded only to the most satisfactory theories. Much benefit has been derived even from the most visionary productions of the human mind: facts have thus been collected, their mutual relations ascertained, and their applications pointed out; which had previously appeared to be isolated, and incapable of contributing to the progress of science: we have thus risen from partial to general views, and consequently made important additions to our knowledge. Much is due to those daring investigators who have ventured to traverse the shades of obscurity without a guide: we have often seen them strike a spark in their course, from the objects they have encountered, that has been caught by the genius of Physics to light up the torch of Experience.

ANATOMY (more decidedly, perhaps, than any other subject of our enquiries,) evinces the difficulties which attend the acquisition of knowledge, even of those objects the most evident to our senses. The investigation of it has been pursued with a certain degree of assiduity for nearly three centuries, and much important information respecting it yet remains to be acquired. After the spirit for original research had languished for a considerable period throughout the greater part of Europe, it revived with ardour in the illustrious VICQ-D'AZYR and BICHAT; and in the latter, in particular, guided by his original and penetrative views, promised to advance it with rapidity to perfection. * * * The spirit he roused in his contemporaries is, however, not extinct; and we may observe, in particular, that the investigation of one of the objects especially pointed out by his doctrines—the ganglionic system of nerves,—is now pursued, in Germany especially, with all the energy we could desire. The most interesting accounts that have been published of labours on this subject lately instituted, are those of WEBER, on the *comparative anatomy of the sympathetic nerve*;* and BOCK on the *fifth pair of nerves and their anastomoses with other nerves, particularly those of the ganglion system.*† But

* *Anatomia comparata Nervi Sympathetici. Auctori* E. H. WEBER. Lipsiæ, 1817.

† *Beschreibung des fuenften Nervenpaares und seiner Verbindungen, mit anderen Nerven, vorzueglich mit dem Gangliensysteme.* Von D. AUG. CARL BOCK, Prosec. an den Anatom. Theatre zu Leipzig. Meissen, 1817.

the date of the period of the publication of those works is beyond that within the limits of which our particular observations must be confined. We have, however, to notice an interesting enquiry of Dr. HIPPOLYTUS CLOQUET, respecting the *ganglions of the nasal fossa, and their nervous communications.**

Anatomists have varied in their opinions respecting the existence of a communication between the nasal cavity and the palate, by means of the anterior palatine foramen; and whether the pituitary membrane lined such a cavity, or was reflected over its orifice. VIDUS-VIDIUS, SPIGELIUS, STENON, VERRHEYEN, KULM, RUYSCH, DUVERNEY, and SANTORINI, admit the existence of membranous canals, forming a communication between the palate and nasal fossa, which the first anatomists of the eighteenth and nineteenth centuries, as BERTIN, LIEUTAUD, HEISTER, HALLER, PORTAL, SCARPA, and BOYER, have sought for in vain. ALBINUS, WINSLOW, and BICHAT, do not speak of them. M.M. JACOBSON and CUVIER, in a Memoir lately read to the Academy of Sciences, deny its existence; but M. Cuvier admits that it is to be found in the other *mammiferi*, with the exception of the horse; and that, in herbivorous animals particularly, the cavity contiguous to the nasal fossa was occupied by a peculiar organ receiving a considerable number of nervous filaments, apparently appertaining to some faculty which man probably does not possess, by which they distinguish poisonous plants from those adapted for their use. The reflections such a presumption gives rise to forcibly struck Dr. Cloquet, and led him to examine, with much accuracy, numerous heads of man and various other animals, with the view to determine the question respecting those in which such an organ exists, and to trace its nervous communications. The following is an account of the results of his investigations, as regards the human species:—

In the middle of the anterior palatine canal there is a small reddish-coloured fungous, and somewhat fibro-cartilaginous, mass; of an ovoid form, surrounded by a fatty cellular tissue: in reality, a nervous ganglion; the large extremity of which sends to the spheno-palatine ganglion of MECKEL two nervous filaments, which were discovered by COTUGNO, and termed by SCARPA the naso-palatine nerves. The small extremity of this ganglion gives off one or two filaments, which are transmitted by two particular canals to the arch of the palate, where they ramify and anastomose with branches of the palatine nerve.

---

* *Nouveau Journal de Medecine,* tome ii.

This little ganglion, termed by Dr. Cloquet *naso-palatine*, from the distribution of its nervous filaments, has then a double communication with the ganglion of Meckel; the one by the naso-palatine nerve, the other by means of the proper palatine nerve. The communications of the naso-palatine, with the spheno-palatine, ganglion, being thus established, Dr. Cloquet endeavoured to confirm, by more particular observations, the existence of the latter, and to show its connexion with the other nervous ganglions of the head. This ganglion, first described by Meckel,* is placed a little without the spheno-palatine foramen: it is of a reddish colour, rather firm in consistence; of a triangular or cordiform shape; variable in bulk, but always inconsiderable; convex on its external, and flattened on its internal surface; and so embedded in the adipose cellular tissue of the pterygo-maxillary sinus, and so confined between the bones, that the preparation of it requires considerable precaution and address. Few persons have been so fortunate as to discover it. Bichât himself† was led to believe that it was merely a nervous enlargement from which secondary filaments emanated.

It is this ganglion which supplies the pituitary membrane with the greater part of its nerves, the number of which varies at their origin: Meckel counted three or four; Dr. Cloquet has seen five: they penetrate immediately into the nasal fossa, by the spheno-palatine foramen. It also sends branches to the palate, and to the parts in the region of the pharynx.

From the different communications of the nerves of the mouth with those of the nasal fossa, observes Dr. C., we may, perhaps, be allowed to conclude that the naso-palatine ganglion contributes to produce the sympathetic phenomena which exist between the senses of smell and taste; and that it also explains, to a certain degree, the mode in which many substances applied to the palate act on the pituitary membrane of the nasal cavity.

The retraction of the testicles, and painful pressure of them against the abdominal ring, and the spontaneous reduction of hernias, occasionally witnessed, and not satisfactorily explained by our anatomical knowledge of the parts thus engaged; induced Dr. JULIUS CLOQUET to investigate the origin and development of the *cremaster muscle* with more accuracy than appears to have been previously em-

* *De Quinto Pare Nervorum Cereb.* sect. iii. & liv.

† *Anat. Descriptive,* iii. p. 174.

ployed on this subject. We shall relate the facts he has discovered, without entering into the particular consideration of the account he has published of the course of his investigation.*

The cremaster muscle is formed from the inferior fibres of the internal oblique, which are drawn without the external abdominal ring by the gubernaculum and testicle, to which they adhere from the time of the descent of the latter; in the same manner that extensible cords, fixed at their two extremities, would give way when drawn by their middle part. In the greater number of instances, the testicle, in descending, passes simply below the inferior border of the internal oblique, aponeurotic fibres of which pass behind the internal margin of the ring, and are inserted into the pubis; these fibres are stretched by the testicle, and lengthened as it descends, forming layers of fibres passing before, and being folded back again on the whole posterior surface of the testicle and spermatic cord. In other instances, on the contrary, the testicle evidently traverses the fleshy fibres of the muscle; since layers of them are observed, not only before, but also behind, the testicle and spermatic cord. The external layer of fibres of the cremaster is almost constantly more voluminous than the internal; sometimes they are equal in bulk, and in a few instances the internal is most developed. In some cases, the internal layer does not appear to exist. This arises from the following causes:—the fleshy fibres forming the inferior margin of the internal oblique are inserted into the pubis by means of fine aponeurotic fibres, which vary in length. If the latter are short, they remain concealed behind the inner pillar of the ring, and the correspondent fasciculus of the cremaster is very visible, and appears fleshy from its origin: if, on the contrary, they are very long, this same fasciculus appears, at first view, to be wanting; but, if the spermatic cord be drawn from below upward, we may perceive very delicate aponeurotic fibres, which, in the form of whitish fasciculi, arise and diverge from behind the internal pillar of the ring, gradually increasing in bulk, becoming more and more red, and at length turning upwards from behind the testicle to be continued to the external fasciculus, which is always evidently muscular. The cremaster muscle, thus produced, forms a sort of sheath, the fibres of which unite at the superior part in two triangular fasciæ; one of which is continuous with the external, the other with the internal, margin of the ring. This may be placed in another point

---

* *Nouveau Journal de Médecine*, tome ii.

of view, regarding the external fasciculus as the origin of the muscle, the fibres of which, after having been spread out in descending over the anterior surface of the spermatic cord and testicle, become re-united on passing over the internal surface of those parts, and are inserted into the pubis.

From specimens of the ethmoid and sphenoid bones, prepared by the late Dr. CASPAR WISTAR, of Philadelphia, it appears, that the generally-received account of the formation of the *sphenoidal sinuses* is not strictly correct.

"It had been long believed," observes Dr. Wistar, "that the sinuses, or cavities in the body of the os sphenoides, were exclusively formed by that bone, when WINSLOW suggested that a small portion of the orbitar processes of the ossa palati contributed to their formation.* Many years after Winslow's publication, BERTIN described two bones which form the anterior sides of those sinuses, and contain the foramina by which they communicate with the nose.† These bones he denominates 'cornets sphenoidaux,' and states that they are most perfect and distinct between the ages of four years and of twenty; that they are not completely formed before this period, and that after it they appear like a part of the sphenoidal bone. According to his account, they are laminæ of a triangular form, and are originally in contact with the anterior and inferior surface of the body of the os sphenoides; so that they form a portion of the surface of the cavity of the nose. He believed that, as they increase in size, they become convex and concave, and present their concave surfaces to the body of the sphenoidal, which also becomes concave, and presents its concavity to those bones; thus forming the sinuses. This account of Bertin has been adopted by SABATIER, and also by BOYER, who has improved it by the additional observation, that these triangular bones are sometimes united to the ethmoid, and remain attached to that bone when it is separated from the os sphenoides. BICHAT and FIFE have confirmed the description of Boyer. Some specimens of ethmoid and sphenoid bones, which I have prepared, will demonstrate that, in certain subjects, about two years of age, there are continued from the posterior part of the cribiform plate of the ethmoid bone, two hollow triangular pyramids, which, when in their proper situation, receive between them the azygos process of the os sphenoides. The internal side of each of those pyramids applies to the aforesaid azygos

* *Memoires de l'Académie des Sciences,* an. 1720.

† *Op. cit.* an. 1744.

process: the lower side of each forms part of the upper surface of the posterior traces; the external side, at its basis, is in contact with the orbitar process of the os palati. The base of each pyramid forms also a part of the surface of the posterior traces, and contains a foramen which is ultimately the opening into the sphenoidal sinus of that side. In the sphenoidal bones which belong to such ethmoids as are above described, there are no cells or sinuses; for the pyramids of the ethmoid bones occupy their places. The azygos process, which is to become the future septum between the sinuses, is remarkably thick, but there are no cavities or sinuses in it. The sides of the pyramids which are in contact with this process are extremely thin, and sometimes have irregular foramina in them, as if their osseous substance had been partially absorbed. That part of the external side of the pyramid which is in contact with the orbitar process of the os palati is also thin, and sometimes has an irregular foramen, which communicates with the cells of the aforesaid orbitar process. Upon comparing these perfect specimens of the ethmoid and sphenoidal bones of the subject about two years of age, with the os sphenoides of a young subject who was more advanced in years, it appears probable that the azygos process, and the sides of the pyramid applied to it, are so changed in the progress of life, that they simply constitute the septum between the sinuses; that the external side of the pyramid is also abolished; and that the front side and the basis of the pyramid only remain; constituting the cornets sphenoideaux of Bertin."*

There is no subject in medical science on which greater diversity of opinion prevails, than on the utility of PATHOLOGICAL ANATOMY. By some it has been considered as the only solid foundation for the practice of medicine; whilst others have thought that it can contribute but little either to our knowledge of the real nature of disease, or of the means for its relief. On this, as on all other questions which depend in a great measure on opinion, we must equally endeavour to avoid having our views confined by those which persons not well-informed on the subject are enabled to take, as to prevent our judgment being deluded by that bias which all men have towards their favourite pursuits. As objections to it, it has been said, that we often cannot detect organic injury after the existence of many of the most violent of diseases, and when it was most strongly suspected to exist;

* *Transactions of the Philosophical Society of Philadelphia.* Philadelphia, 1818.

whilst, on the other hand, great and extensive change of structure have been witnessed in organs, the integrity of which has been considered most necessary to health, when no apparent derangement of functions, either local or general, had been observed. These objections are to a certain degree valid, but not to the extent the proposers of them would insinuate: they are not applicable to pathological anatomy, when it is studied with those views which have guided its most eminent cultivators in the latter part of the last and during the present century; views which regard organic injury as the consequence of one series of morbid actions, but which will itself become a cause of others. Of the latter class of diseases, it will furnish the most decisive and important knowledge; provided it be also considered, that, as organic and functional derangement will be combined, so there will be symptoms present dependent on each of those causes. Of the diseases from mere functional derangement, it will afford nothing more than negative information.* When contemplated in this manner, pathological anatomy has been considered as the most authentic source; whence the description of diseases, and the history of their progress, might be deduced; whilst we remain ignorant of the nature of the *vital principle*, and its mode of action in the state of health,—or, if that term be objected to, of the cause why certain combinations of matter produce certain modes of action,—it cannot possibly be supposed that we can understand the nature of their deviations from the healthy state; and, therefore, the *signs* of such deviations must be the objects whence our precise ideas of them must be derived, and on which all our real knowledge of them must depend.—But we are digressing too far from our professed object.

Until a late period it was generally supposed that extravasation of blood in the brain must necessarily prove fatal, or at least be productive of irremediable disease; for that absorption of it could not be effected by the vessels of that organ. The accurate and extensive examinations made with the view to ascertain this point, by MM. Riobé, Rouchoux, and Cruvelhier, showed the erroneousness of that

* We employ the term *functional* derangement, although it may be objected to by such physiologists as believe that all the varieties of vital action absolutely depend on peculiarity of organization; and, therefore, that alteration of structure must have preceded change of mode of action. It must, however, be considered prudent to admit the use of that term, in the present state of our knowledge, as applied to disease which is not accompanied with, or does not leave, any evident morbid change of structure.

opinion, by the evidence of facts which must be considered as among the most interesting with which pathological anatomy has furnished us. Dr. BRICHETEAU, in a Memoir lately published on *Apoplexy*,* relates an account of several analogous facts that have occurred to his observation. It is the formation of a cyst in the substance of the brain surrounding the coagulum of blood, and the consequent absorption of it by the vessels of the cyst to which we allude. The accurate knowledge acquired on this subject by the physicians whom we have named, is, we believe, not generally understood; we shall, therefore, step without the limits of the period comprised in this sketch, to adduce the description given of those cysts by M. CRUVELHIER, in his Essay on Pathological Anatomy: it is traced after a very extensive series of observations made at the Hôtel-Dieu by himself, and by other anatomists in the different hospitals in Paris.

"On the second or third day following an attack of apoplexy, we find an unequal laceration in the substance of the brain, and a portion of blood, in part fluid, and partly coagulated. Towards the fourth or fifth day, the brain surrounding it presents a yellowish colour, similar to that of the skin and cellular tissue after a bruise. From the ninth or tenth to the fifteenth day, the coagulum is more solid, and is adherent to the surrounding parts, which are red and soft. If those parts are removed in their layers, we find, surrounding the parietes of the cavity containing the coagulum, several successive layers formed of the substances of the brain, and which are covered with red points. No true membrane has yet become formed, but the external layer appears to be the rudiment of it. At a more advanced period, the redness is diminished, and the appearance of a membrane is evident. Lastly, if these individuals are opened one, two, or more, years after an attack of sanguineous apoplexy, we find a cyst, variable in capacity, formed by a very fine membrane, of a yellowish or reddish colour, containing some yellow serum. We must add to this, that, as the blood, serum, &c. are diminished in quantity, the capacity of the cyst becomes reduced; its parietes become thicker, and contract adhesions: its cavity is at length obliterated; it becomes more and more confounded with the substance of the brain; and, at the end of an indeterminate period of time, only a yellowish-coloured cicatrix, or a laminated tissue, sometimes containing a little serum, can be discovered."

We have only to add, that the observations of Dr. Bricheteau

---

* *Journal complémentaire du Dict. des Sciences Medicales,* tome i.

accord exactly with those of Cruvelhier; and that he had, in several instances, found the number of these cysts correspond with the number of attacks of apoplexy which the patient had experienced.

The appearances observed by Dr. Serres in the brain of persons who had experienced paralytic attacks, which we have already noticed,* will be found to correspond with those above described. These observations mutually corroborate each other; particularly as it appears that they have been deduced from investigations undertaken with somewhat different views.

Dr. Bricheteau has also observed several instances of a peculiar disorganization of the structure of the brain after attacks of apoplexy, evidently differing from that described as being discovered around the coagulum of blood preparatory to the formation of the cyst; since it is also found in cases in which death has ensued immediately from the attack; and, from the symptoms which preceded this, it would appear to be the cause, not the consequence, of the apoplectic seizure. We shall detail these symptoms when we consider this subject in a medical point of view.

In one instance, the corpus stratum was found ulcerated throughout great part of its extent: the whole substance of that body was soft, of a greyish colour, and had entirely lost its striated appearance. The portion of the brain surrounding it, to the extent of five or six lines, was of a yellowish colour, and somewhat softer than natural. The remainder of the brain was perfectly healthy, and of considerable firmness of structure. The lateral ventricles contained a small quantity of serum.

In another case, the pia mater and the arachnoid membrane were much injected with blood; the latter appeared to be more thick and firm than natural. The cortical portion of the brain was remarkably altered in structure: many of the disorganized portions contained a greyish-coloured semi-fluid matter, without any appearance of sanguineous congestion; in some other parts, on the contrary, a sanious matter, and, as it were, putrid mass, interspersed with drops of blood, was discovered. This disorganization was very extensive, and spread irregularly throughout the whole of the cortical substance of the brain.

The cerebrum, strictly speaking, in one instance, did not exhibit any apparent alteration of structure; there was but a small quantity of serum in the lateral ventricles; but the tuber annulare was entirely changed in its appearance from

---

* *London Medical and Physical Journal*, vol. xl. p. 351.

the natural state;—the superior part of it being reduced to a semi-fluid mass; the inferior was soft, and of a greyish colour. The cerebellum did not show any evident morbid appearance.

An appearance of the brain, similar to that above described, was noticed by Dr. GORDON, in a patient who died from apoplexy in the Edinburgh Infirmary.*

The appearances of the brain of two individuals, who died after an attack of apoplexy exhibiting the usual symptoms, related by Dr. ABERCROMBIE,† were, it seems, similar to those above described.

The subject for pathological investigation, which has engaged most attention during the late period, is the *spinal marrow*; and the interest attached to it has been greater in proportion to the degree in which it had been formerly neglected, by anatomists in general. The discoveries which have been lately made respecting the precise functions of the different parts of the nervous system have given rise to this enquiry; and, from the facts already ascertained, we are led to expect that we shall thence acquire knowledge of the first importance in the practice of medicine. The physiologists most actively engaged in this investigation at the present time are—FRANCK, HARLES, BRERA, RACHETTI, ESQUIROL, SANDERS, COPELAND, ABERCROMBIE, and REID. It is to the latter, however, that we are indebted for the full development of this subject more especially in a theoretical point of view, and as it relates to the production of two of the most severe diseases to which human nature is liable—*tetanus* and *hydrophobia*.‡ Of disease of the spinal marrow being the general cause of tetanus, there does not now exist a doubt; facts tending to confirm it are constantly witnessed. We refer our readers to the cases related in the last volume of our Journal, that have lately occurred to the observation of Prof. Brera and Dr. Reid; and which show the correctness of this opinion in a very decisive manner. We regret that the limits within which our views must at present be confined, do not permit us to enter particularly into the consideration of the interesting Memoirs of RACHETTI,§ BRERA,‖ and HARLES,¶ on this subject. We shall, however,

* *Edinburgh Clinical Reports*, p. 129.

† *Edinburgh Medical and Surgical Journal*, vol. xiv.

‡ See the Critiques in the present Number of this Journal.

§ *Della Struttura, delle Funzioni, e delle Malattie della Midolla Spinale.* Milano, 1817.

‖ *Giornale di Medicina Pratica*, compilato dal Caval. V. L. Brera. Fas. XXIII. ¶ *Ibid.* Fas. XXIV.

notice them in our Foreign Department on a future occasion.

The opinion of Dr. Reid, that disease of the spinal marrow is the cause of the symptoms of hydrophobia, is not equally confirmed by experience. Dr. Reid was led to adopt that opinion rather from reasoning on the nature of the symptoms, than actual observation: but we would ask —do not those symptoms rather indicate a morbid state of the *ganglion system* of nerves?

Effusion of serum into the theca vertebralis, and sheaths of the nerves thence derived, and the other usual signs of inflammation, have been observed by Mr. Sym, in several persons who died of typhous fever.*

Dr. Thomson, of Jamaica, has also published some observations on this subject, which show that inflammation of the spinal marrow is a frequent cause of the *trismus* of infants; at least, that it is so in that climate.

The following appearances were observed on dissection in a boy who died of the usual symptoms of *hydrophobia*, on the seventeenth day after having been bitten by a rabid wolf:†

The membranes of the brain were covered with a thick layer of coagulable lymph, and their vessels gorged with blood; the salivary glands, fauces, pharynx, larynx, trachea, and alimentary canal, as far downwards as the ilium, showed signs of previous inflammation; the stomach was filled with a yellowish-coloured fluid.

The appearances noticed by M. Morelot in the wolf were merely a dryness of the surfaces of the œsophagus, pharynx, and larynx; the viscera of the thorax and abdomen did not exhibit any unusual appearances.

In a rabid dog, who bit several persons at Knightsbridge, the only morbid appearance witnessed was an inflamed state of the stomach and œsophagus, the surfaces of which were covered with glairy mucus.

In a female, aged 42, who died in the infirmary of Edinburgh, after having been affected with diabetes during two years, the sympathetic nerve, on both sides, from its entrance through the diaphragm into the cavity of the abdomen, to its descent into the pelvis, was enlarged to about three or four times the ordinary size; and there was increase in the bulk of the splanchnic nerves to the same extent, from about two inches within the cavity of the chest, until it perforated the

---

* *Edin. Medic. and Surg. Journal,* vol. xiii.; and *London Med. and Phys. Journal,* vol. xl.

† See the present Number of this Journal.

diaphragm. Nothing respecting the history of the disease, sufficiently interesting to merit being detailed, is noticed in the report.*

The accuracy of observation, and disposition to be no longer deluded by erroneous terms, which characterize and direct the scientific investigations of the present period, have given rise to some interesting observations on the cellular cysts, termed hydatids of the uterus, by Prof. MANGILI.†

Although the term hydatid be correctly applicable to the diseased structures in question, yet the erroneous ideas that have been entertained respecting their nature may, perhaps, have arisen from the same appellation having been given to a species of vesicular parasitical animal. Prof. Mangili says, that he himself formerly entertained an idea that these cysts did not differ from the living animals found in various parts of animal bodies, which they somewhat resemble in structure. But, from the consideration that all animals of that species assume a certain and distinct form, and that these vesicular bodies are various in size and appearance, he was led to suppose that they were essentially different. Besides which, he observes, hydatids have a head; but, in the cysts found in the uterus, such a formation cannot be discovered. The former also contain a portion of lymph, which cannot be made to coagulate; while the latter enclose a dense liquor, from which an abundant coagulum may be precipitated. This coagulum is of a light flesh-colour; when placed in spirit of wine, it becomes opaque, in which fluid the lymph contained in hydatids maintains its transparency. Ramifications of blood-vessels are also manifest on the surface of the uterine cysts.

Prof. Mangili endeavours to account for the formation of these cysts in the following manner:—The internal surface of the uterus during pregnancy becomes lined with a villous structure, the villi of which are formed by a continuation of the arteries of the uterus, from which lymph is effused that is absorbed by the external surface of the membranes of the fœtus. This secretion, under ordinary circumstances, only takes place from that part of the uterus in contact with the placenta; but if, from plethora, or causes unknown to us, the whole of the villous surface of the deciduary membrane secrete the same fluid, it is prevented from

---

* *Edinburgh Clinical Reports*, p. 137.

† *Discorso del.* Prof. GIUSEPPE MANGILI: *intorno alle pretese Idatidi Uterine.* Letto in occasione de laurea nel giorno 3 Agosto, 1818, all' Instituto di Pavia.

becoming diffused by the adhesion of the membranes of the fœtus, it consequently distends the villi which secrete it, until at length they assume the appearance which has been considered similar to that of hydatids.

A species of *nævus maternus*, occasionally witnessed, and which is frequently attended with serious results, has been examined with much accuracy by Mr. Wardrop,* which is situated between the skin and subjacent muscles; he has consequently termed it the *subcutaneous nævus*. It usually forms a tumor, which not unfrequently acquires a considerable bulk within a short period after birth. The tumor consists principally of blood-vessels, terminating in a spongy structure, composed of numerous cells and canals of a variety of forms and sizes, which communicate with ramifications of the blood-vessels. These cells and canals have a smooth and polished surface, and in some parts much resemble the cavities of the heart; fibres crossing them in various directions, like the cordæ tendineæ. Several of these vessels, which, from the thinness of their coats, appeared to be veins, were of a large size: in one case, examined ten days after birth, where the tumor was situated on the back part of the neck, there was one sufficiently capacious for the admission of a large-sized bougie.

Some interesting observations made on the examinations of the bodies of several children who had died from the effects of worms in the digestive organs, have been related in a Memoir read to the Medical Society of Paris, by M. Gaultier de Claubry (père).

In a girl, seven years of age, who died after having been affected with convulsions for six days, and who had previously been in good health, M. C. discovered eleven worms of considerable magnitude in the cavity of the peritoneum, lying among the mass of intestines. The stomach was found to be pierced with holes, through which the worms had passed; and several of these animals were then engaged in effecting their passage through its coats.

In another child of about the same age, and who died on the seventh day from an attack of convulsions, thirty-six *lumbrici* were found dispersed among the intestines in the peritoneal cavity; and twenty-seven were found yet working their way out of the stomach. This viscus al-o contained an enormous mass of them, all of which were of the same species. The intestines were distended with air. A considerable quantity

* *Medico-Chirurgical Transactions,* vol. ix. part i.

of serum was found effused on the surface of the brain, and contained in its ventricles.

The extent and accuracy of the observations of Doctor MACARTNEY (the professor of Anatomy at Trinity College, Dublin), and the attention he has paid to pathological anatomy, leads us to attribute much importance to his account of the appearances witnessed in the bodies of persons who had died of the epidemic fever of Dublin. The following summary is drawn up as a reply to a query of Dr. BARKER, —whether they were those of genuine inflammation, or of any peculiar kind?—"Having reviewed my notes," says Dr. M. "on the anatomical examinations of persons who have died of typhous fever, I can state, as the result of my experience, that the morbid appearances are not those of common visceral inflammation. A great proportion of the subjects for anatomical lectures in Trinity College, during last winter, appeared to have been of the present epidemic, as they had petechiæ on the surface; and my late observations on these have enabled me to confirm the above conclusion, which I had adduced many years since. The morbid appearances that strictly belong to typhus are the following, accordingly as the head, lungs, or abdominal viscera, are engaged in the disease:—1st. Fullness or distention of the vessels of the brain, especially the veins; some water effused on the surface and into the cavities of the brain. 2d. The same species of congestion in the lungs, and different degrees of effusion in the cavities of the pericardium and pleura. 3d. Venous congestion in the liver, spleen, or alimentary canal; sometimes a blood-shot appearance, or spots of extravasation, in the mucous coat, more particularly in the stomach and first coils of the intestines. In some instances, a more generally pulpy, or swollen, and discoloured state of the mucous coat of the alimentary canal. These congestions were always of a purple or venous colour; and the blood throughout the body appeared to be accumulated in the venous system, and had little tendency to coagulate. Such were the appearances attendant on the congestions observable after typhous fever.

"The morbid appearances in real and pure inflammation are these:—1st, in the head.—The minute branches of the arteries appear more numerous than usual, from carrying florid red blood. The effusion which takes place is more consistent than in the former case, and appears like whey; or pus is secreted on the membranes; the arachnoid coat is thickened and opaque. 2d. In pleuritis and pericarditis, there is the same distribution of the arteries, and a wheyishlooking fluid, pus, or lymph, thrown out. In inflammation of the substance of the lungs, there is always venous congestion; but the small arteries also are increased, and the lungs feel more firm than in typhus. 3dly. In gastritis and enteritis, the inflamed parts are denser; the redness is brighter than in typhus. The peritoneum is liable to be involved, and the termination is slough or ulcer after a certain time. In cases where general fever is combined with real local inflammation, as sometimes occurs in dysentery, or when pneumonia is combined with typhus, or the latter with permanent and violent delirium, the peculiar morbid appearances of each disease are to be observed in combination.

Two facts deserve to be recollected:—1st. That the duration of general fever and visceral inflammation is not the same. 2d. That internal inflammations are very common in hot-blooded animals, but idiopathic fever is peculiar to the human kind. It may be added, that processes of an inflammatory nature are fitted for repairing parts that have their functions interrupted or their structure injured; but the effects of typhous fever have no such power."*

We have hitherto passed over unnoticed what, if utility be the standard by which the value of knowledge is to be estimated, must be considered the most important acquirements in pathology that have been made during a long time past: it is those of Mr. Brodie, respecting diseases of the joints, to which we allude.† We cannot, however, treat of them on the present occasion: the conciseness of observation which the limits of our plan oblige us to adopt could not convey an appropriate idea of them to our readers; besides which, we presume that no surgeon can be uninformed of the opinions of Mr. Brodie on this subject, which were

---

* *Transactions of the Fellows and Licentiates of the College of Physicians in Dublin*, vol. ii. We have just received this work, which contains much other interesting matter respecting pathological anatomy; but the lateness of the period, and previous arrangement of the greater part of our historical observations for the press, prevent our entering into the consideration of it on the present occasion. Such observations as may be illustrative of any subject of particular interest, as a hasty perusal of it may afford, shall, however, be adduced. We may here observe, that this explanation will apply to several other medical works which have just appeared; some of which, from the character of their authors, doubtless contain matter that will add much to our knowledge.

† *Pathological and Surgical Observations on Diseases of the Joints*, by B. C. Brodie, F.R.S. assistant Surgeon to St. George's Hospital, &c. &c.

published some years since in the Transactions of the Medico-Chirurgical Society. Those we mentioned are the development of these views, matured by additional experience and reflection. We shall make our readers better acquainted with them at a future period.

Some important addition has been made to our knowledge of the organic pathology of the heart, by M. LE BARON CORVISART, in a third edition of his work on diseases of that organ.* To particularize facts, however, does not come within the scope of our plan on the present occasion, as they principally consist in observations which have confirmed what has been described by others, some of which had been noticed in the former editions of the work in which they appear; or of reflections which necessarily arise from the consideration of facts previously ascertained. For some interesting information on this subject, we refer our readers to the Memoirs of M. ROSTAN and Dr. MOTT, in the last volume of this Journal.

We felt disposed to preface the relation of the recently-discovered facts in PHYSIOLOGY,† with a general view of the circumstances which have rendered that beautiful science what it is at the present period; but we could not do this without assigning to the labours of one of the philosophers to whom we should refer, (who had not, it is true, the eloquence of a CABANIS, the vivid imagination of a BICHAT, nor the erudition of men whose names we do not mention, because they might witness our attempt to describe their merit; and, therefore, the precision of his views, the extent of his observations, and the depth of his knowledge, are, to a certain extent, hidden in obscurity;) a degree of importance that is not generally conceded to them. We therefore neglect such an attempt, and shall proceed immediately to the consideration of facts, the particular observation and application of which are due to the development of

---

* *Essai sur les Maladies et les Lesions organiques du Cœur et des gros Vaisseau.* Paris, 1818.

† We shall employ this term in its real and most comprehensive sense, as far as regards the functions of animal bodies. Indeed, the more we reflect on this subject, the more unable we feel to make a distinction between these actions, in health and in disease, in our enquiries into their nature and causes. That modification of it, therefore, termed *pathology*, will be embraced in the same point of view. Evident altered form of structure has been already distinctly considered.

that mode of pursuing the study of this branch of science, which was instituted in the latter part of the last century. It is the phenomena observed by Dr LALLEMAND in a fœtus born without brain or spinal marrow, to which we at present refer.*

The doctrine of *irritability*, of the illustrious HALLER, appears to be at length almost universally abandoned: a doctrine which continued to excite disputes among the most eminent physiologists of Europe, for a considerable period after those immediately educated in his school had ceased to exist. Whether the opinions of PROCHASKA† and SCARPA,‡ respecting the local production of the nervous influence through the whole system of nerves; of WINSLOW§ and BICHAT‖, and their followers; or of LEGALLOIS¶ and PHILIP,** respecting the functions of the ganglion system of nerves be admitted, it is immaterial: the phenomena on which Haller founded his theory of muscular irritability, may be better explained by the application of facts, respecting which there is neither doubt nor diversity of opinion:—the power of the ganglion system to retain within itself, for a certain length of time, after having been separated from the rest of the nervous system, a certain degree of nervous influence; and the distribution of the nerves of that system over every part of the body, by means of their connexion with those of the brain and spinal marrow.

The facts related by Dr. LALLEMAND powerfully support the opinion of Winslow and Bichât,—that of the independence of the ganglion system on the brain or spinal marrow for its nervous influence; the continuance of the action of the heart and of all the functions of organic life, after the entire destruction of the latter sources of nervous influence, cannot well be explained in any other way. It may be said, that the blood returned from the mother by the veins of the placenta was a sufficient stimulus to the heart for the support

---

* *Observations Pathologiques propres à éclairer plusieurs points de Physiologie*, &c. and *London Medical and Physical Journal*, vol. 40, p. 343 et sequent.

† *Commentatio de Functionibus Systematis Nervosi*, published in 1784, in the third fasciculus of the *Annotationes Academ.* of that author, and reprinted in his *Opera Minora*, at Vienna, 1800.

‡ *Tabulæ Neurologicæ ad illustrandum historiam Anatomicam Cardiacorum Nervorum*, &c. Ficini, 1794.

§ *Exposit. Anatom. Traité des Nerfs*, § 364.

‖ *Recherches Physiologiques sur la Vie et la Mort.*

¶ *Expériences sur le Principe de la Vie.*

** *Experimental Enquiry into the Laws of the Vital Functions.*

of its action; but this will not satisfactorily account for the continuance of nutrition, and the development of the structure of the fœtus, which supposes the presence of nervous influence, although we are obliged to admit the development of the embryo to a certain extent before the existence of nerves. To compare the life of the fœtus in the uterus to that of a vegetable, and thence argue the possibility of its growth without nervous influence, appears to be a forced and untenable proposition, and to be satisfactorily refuted by the voluntary motions which the fœtus is known to execute while in the uterus, under ordinary circumstances, as well as in the case which forms the particular subject for consideration at present,—motions appertaining to animal, not to organic, life. We do, however, perceive one way by which these facts may be otherwise explained—by supposing that there may be a continuation of nerves from the mother to the fœtus along the course of the umbilical cord: this appears to us to be an interesting subject for enquiry, but which would be attended with many difficulties, and require great accuracy in its investigation.

The cessation of the action of the heart on destruction of a considerable portion of the brain or spinal marrow, which was observed in the experiments of M. Legallois, and which has been adduced to refute the opinion of the distinctness of the nervous power of the ganglion system, is explained by Dr. Lallemand, by the shock received by the latter system from its sympathetic connexion with the former; which sympathy M. Legallois and Dr. Wilson Philip have shown to become more intimate in proportion to the greater length of time from the period of birth. The experiments of that gentleman, and those of Dr. Philip, prove, that injury to the brain and spinal marrow of a young animal does not so certainly and immediately stop the action of the heart, as a similar injury of those parts in an older one. It appears also, from the nineteenth, twentieth, twenty-first, and twenty-second, experiments of Dr. Philip, that, if the injury of the brain or spinal marrow has not been sufficient to cause the entire cessation of the action of the heart, but only to disturb and debilitate it, that it will recover its former energy to a certain degree, after the lapse of some time; that is, when the nervous system supplying it has recovered from the shock it had experienced.

A question then arises,—to what cause is the death of the organic system to be attributed, when the influence of the brain on that system is abolished? This is explained by Bichât, from the cessation of respiration, which is dependant on the influence of the brain, and which is the connecting link between the systems of animal and organic life, becoming after birth equally necessary to both.

The influence of respiration in this respect is well explained by the opinion of Dr. Broussais,* that the action of red blood on the nerves is necessary for the continuance of their influence, and that the motions of the heart cease after birth, if respiration be not continued, from the loss of that influence. But what is it, observes Dr. Broussais, which, in a fœtus without brain and medulla oblongata, prevents the existence of respiration?—it has all the organs immediately necessary for the actions which constitute that function; and, since it had the power of moving its limbs when in the uterus, why could it not also move the diaphragm and other respiratory organs, to procure the air it stood in need of?—because, he replies, the nerves and muscles, although well formed, did not communicate with a common centre.

Legallois stated, that the diaphragmatic and all the other nerves of the muscles which produce the mechanical phenomena of respiration, take their origin in the spinal marrow, the same as the other nerves of the trunk. How is it, then, that after decapitation the respiratory motions are abolished, and all the other motions of the body exist? This, Legallois observed, was to him one of the greatest mysteries of the nervous system,—a mystery which he thought would sooner or later be developed, and the discovery of which would throw an important light on the mechanism of the functions of this wonderful power. Some facts, he continues to observe, witnessed in the course of his experiments, led him to believe that the accessory nerve of the eighth pair performs a principal part in the dependance of respiration on the brain.† This mystery Dr. Broussais would explain in the following manner:—

The dilatation of the thorax is determined by the sensation excited by the necessity of respiration, which sensation resides in the mucous membrane of the pulmonary organs: it is transmitted to the brain by the eighth pair of nerves, and is received by that organ at the superior part of the medulla oblongata, where those nerves terminate; and it is thence the volition arises which puts in motion the respiratory muscles. But it is not by the nerves of the eighth pair, which brought the sensation, that the volition is transmitted;—it runs along the spinal marrow, and thence in the cervical nerves which go to the respiratory muscles.

---

* *Journal Universel des Sciences Medicales*, tom. xii.

† *Expériences sur le principe de la Vie*, p. 36 and note.

Now, if the central point where the eighth pair of nerves terminate, be destroyed, the necessity for respiration is not felt. If, on the other hand, the spinal marrow be divided between that part and the origin of the nerves we mentioned, the necessity for respiration is felt; but, from the interruption to the course of volition, respiration is impeded. An animal on whom the division of the spinal marrow has been thus effected, continues to make some vague attempts at inspiration, and a deranged kind of respiration is carried on for a short time; but this is effected through the medium of branches of the fifth pair of nerves.

Dr. Broussais continues to observe, that it may be asked,—how is it that the muscles of the trunk, which do not serve for respiration, are not equally affected by the division of the spinal marrow? These muscles, the author replies, are so far influenced, that, although they may perform motion, it is not voluntary motion. Dr. B. also thinks that the muscles which should dilate the thorax still retain some power to do so, but that it is not equal to the force which opposes that action.

Supposing the motions of the fœtus which the mother stated to have experienced in the case related by Dr. Lallemand, to have really taken place, it would establish a physiological fact of great importance, and which could not be satisfactorily demonstrated in any other way,—that the ganglion system may act on the nerves of the muscles of voluntary motion without the intervention of a common centre. Such an influence of the ganglion on the cerebral system of nerves, will explain many curious physiological and pathological facts daily witnessed. Bichât thought that the movements of a person asleep, labouring under indigestion, arose from the influence of the ganglion system on the muscles of voluntary motion through the medium of the brain; which medium it is necessary, in this and other circumstances under which it is witnessed, to admit; and, although the exertion of voluntary power may not be evident, the continuance of respiration shows that such a power exists. Such are the motions observed in apoplexy, epilepsy, and catalepsy, concussion of the brain, &c. and, perhaps, in hysteria. Dr. Broussais thinks that many morbid appearances in various parts, which accompany inflammation of some one of the abdominal viscera, may be explained by the influence of the ganglion system of nerves, without the intervention of the spinal and cerebral system: such are—redness of the extremity of the tongue, and the conjunctive membrane of the eye; and, particularly, dryness and burning heat of the skin, in inflammation of the mucous membrane of the intestines; while other sympathetic affections produced by the same disease,—such as tenderness of the limbs, and the muscular fibres of the anterior parts of the body especially, prostration of strength, thirst, cephalalgia, despondency, and delirium,—may be attributed to the association of the digestive organs with the cerebral system, and the whole of the organs of animal life.

These circumstances Dr. Lallemand thinks will explain why, in cases of paralysis, when sensation and motion are not entirely abolished, the inferior extremities are less affected than the superior; and that, when the recovery of those functions takes place, it is first manifested in the inferior extremities. He speaks of having witnessed an instance where the arms were paralytic, the voice embarrassed, and the ideas confused; at the same time that the patient could walk tolerably well: on opening the body, in this case, serous effusion was found in the lateral ventricles of the brain. And, in cases of inflammation of the arachnoid membrane, he has witnessed the convulsive motions more violent in the arms than in the legs, and often exclusively confined to the former.

Such facts are curious, and appear to show that the spinal marrow does not receive its power of action from the brain; besides which, the facts observed in the acephalous fœtus prove that one system of nerves, at least, do not derive their influence either from the brain or spinal marrow. The whole series of facts render probable the opinion of Prochaska and Scarpa, that the power of producing the nervous influence, both in those of organic and animal life, resides in the nerves themselves; and that their communications with a common centre, as the brain, is only to establish a mutual relation between them. If we admit the opinion of Dr. Broussais, that the presence of red blood is necessary to the production of this nervous influence, there seems to be nothing wanting for a satisfactory explanation of the phenomena which we have noticed.

There are some observations of Dr. Broussais on the functions of the brain, which we must not pass over without attention, as they relate to some important pathological phenomena which are daily witnessed:—the existence of the functions which are generally considered to depend on the influence of the brain, at the same time that the power of voluntary motion seems abolished.

Whatever may be the nature of the sentient principle, observes Dr. Broussais, it is in the encephalic centre that consciousness, or the faculty of perceiving one's own existence, resides, and by which a person distinguishes himself from any other individual; and it is from the same part that the determinations which induce motion are transmitted: but it does not thence follow, that all the impressions which arrive at the encephalic centre are perceived by the principle before mentioned, or are sensations; nor that the will orders and determines all the motions which depend on the influence of that centre: for instance,—the fœtus has neither sensation nor will, and yet impressions, which would be qualified by the term affections or sensations, arrive at the brain, and determine motions of the muscles of the body generally, which would be called voluntary if it were born and awake. The apoplectic and epileptic are in the same state: when pinched, they contract muscles, the motion of which depends on the brain; but can it here be said, that the individuals have felt or suffered, or acted voluntarily? The correspondence, therefore, between the senses (at least those of feeling, either external or internal,) and the cerebral centre, and between this centre and the loco-motive muscles, commences before the development of consciousness, and continues during life, when consciousness does not give any proof of its existence in the individual.

The faculty of perceiving one's own existence, to will and to execute with consciousness, is, then, a faculty which may or may not exist, without our being permitted to infer from its absence that impressions, and consequent motions, may not follow their usual course, and be executed in the usual manner: and thus it is that respiration may be carried on through the influence of the eighth pair of nerves, under circumstances where voluntary power is apparently abolished.

We must, therefore, according to the doctrine of M. Broussais, make a distinction between sensibility and excitability of the brain,—between sensation and the power of volition.

"If," as M. Castel well observes,* "the will be a desire applied to a certain object to obtain a certain end it centres in the dominion of the intellectual faculties, can we suppose that this dominion exists in a segment of the spinal marrow which does not communicate with the brain?" (the experiments of Legallois show that irritation of a portion thus separated will excite action in those muscles of voluntary motion which receive their nerves from that portion.)—

---

* *Journal complémentaire du Dictionaire des Sciences Medicales,* tome 1, p. 199.

"Without doubt, a degree of excitability remains in this segment; but there is a great difference between sensation and volition: it cannot be a centre of sensation; it is, at the utmost, only a centre of irritability."

The general conclusions deduced by Dr. Broussais, from the facts we have related, and others, apparently of less importance, which our limits will not permit us to enter into the consideration of, are—that the nerves throughout their whole extent possess in themselves their power and their properties; that they do not derive these from the brain, and only communicate with it for the support of a mutual relation between the different organs of the body; that the nerves may be divided into two orders,—the one adapted for the reception of external impressions, the centre of which is in the superior part of the medulla oblongata,—the other for mutual relations of this centre with the internal viscera, among which it has its own peculiar centres; and that the relation between these two orders of nerves is established in the following manner:—the encephalic centre receives sensations from all parts of the body, but it can produce motion in a direct manner only in the muscles which are attached to the skeleton: it has no influence on the motions of the muscles and tissues of the viscera, except through the medium of the ganglion system. The nerves of the viscera produce sensations in the encephalic centre, and may force it to produce in the muscles dependent on its influence the motions which it may require; but they cannot cause the production of them in a direct manner, at least, not after birth.

We shall now take a general view of the illustrations, which the application of our knowledge of the preceding facts affords of several other physiological and pathological phenomena, particularly those observed in the functions of some of the thoracic and abdominal viscera. In doing this, we shall adopt the reflections of the author of the *Histoire des Phlegmasies Chroniques.*

The influence of the nerves of the ganglion system, observes Dr. Broussais, on those which serve for the production of voluntary motion, will explain that association of action which is witnessed between the thoracic and abdominal viscera and the muscles attached to the skeleton that surround those organs: this association is so strict, that, with the exception of the heart, no motion of any importance occurs in any of the organs of those two cavities, without being accompanied with another in the muscles which have been mentioned. It is easy to understand that, since the action of those viscera are independent of the brain; since there is a communication of action between them independent of its influence; and since they have the power to force that organ to render them assistance on certain occasions,—that all these actions can only take place through the medium of the ganglion system of nerves. Various pathological phenomena arise from diminished sensibility of the brain to the appeal thus made to it. Thus, in the apoplectic, vomiting will often not take place until four or five times the quantity of tartar emetic ordinarily necessary to produce that effect, has been exhibited. What does this show?—not that the stomach is paralyzed, since it continues to digest the food and pass the chyle into the intestines as usual; but that the eighth pair of nerves, which make the appeal to the brain to obtain the contraction of the diaphragm which is a necessary part of the action of vomiting, make less impression on that organ than usual, in consequence of the compression which it suffers. The stertorous respiration that is observed in the same disease, that which appears in a person in great agony, and the slowness of that action in deep sleep, may be referred to the same laws. The same laws will explain the retention of urine and fæces of apoplectics. We know that the expulsion of those excrements does not occur, under ordinary circumstances, particularly that of the latter, until the cerebral centre, informed of its necessity, communicates an increase of power to the muscles of the bladder and rectum, by means of the nerves under its dominion.

We immediately perceive, that, if the nerves of the ganglion system possessed the power to command the action of the muscles of the skeleton without the mediation of the cerebral centre, that they would in these cases produce it, and oblige them to act with energy; just as they force the heart to rapidity of contraction, in cases where inflammation of the thoracic or abdominal viscera is combined with apoplexy. This is the state which is observed in cases of severe peripneumonia and gastro-enteric fever, where the pulse and all the actions of the vascular system are increased, although cerebral congestion prevents the free action of the muscles of the skeleton, and produces stertorous breathing, constipation, and retention of the urine.

It is, therefore, evident that the ganglion system has no influence on the muscles of the skeleton after birth, without the concurrence of the cerebral centre; and that it exerts, on the contrary, an absolute empire over the heart and the whole vascular system. We also perceive why the muscles of the extremities are less dependent on its influence than those of respiration: it is because it sends but a few small branches to the great trunk of the nerves of the former parts, while it furnishes to the latter, nerves which equal in bulk those of the other system. If we examine this subject attentively, it will be also evident that the influence of the ganglion system on the different structures of the human body is in a direct ratio with its material predominance.

In observing the apoplectic, in whom *animal life** is much diminished, we have remarked the continuance, and sometimes an increase, of the *actions of organic life** which appertain to the heart and system of capillary blood-vessels. This absolutely inverse action observed in the phenomena of the two divisions of life, obliges us to conclude that the *organic* is particularly under the influence of the ganglion system. It would be an interesting enquiry to ascertain to what degree it is submitted to the influence of this system, and to ascertain, if possible, the influence the cerebral centre and the nerves of animal life are capable of exercising over the phenomena which constitute it.

Dr. Broussais considers that assimilation and nutrition are functions which should strictly be excluded from its dominion: for that in plants and zoöphytes, which have no nerves,—assimilation, nutrition, increase, and decomposition, take place. In the higher classes of animals, which are provided with nerves, since nutrition precedes the development of nerves,—since, after having formed them, it continues to support them during the remainder of life,—it is evident that this function is not to be attributed to them. This function is dependent on *vital chemistry*, to ascertain the nature of which it would be first necessary to ascertain the nature of the principle of life. Vital chemistry is, therefore, the first of the phenomena of animalization.

Although vital chemistry is not the direct effect of the nervous influence, it does not thence result that this does not exert on it any kind of influence: this is the precise point on which the obscurity attending this question depends. It may be considered that the nerves influence nutrition, not in producing themselves the compositions and decompositions of vital chemistry, but in supplying to the different tissues of the body the materials necessary for the phenomena of its operations; that is to say, by the motions which they determine in the animated machine,—motions without which the supply of materials for nutrition could not take place. Thus we find that the sensation arising from the want of those materials induces a new or more

* We employ these terms strictly according to the doctrine of Bichât.

copious supply of them, producing an increased afflux of blood to the part. This important office of the visceral nerves appears with particular evidence in the sympathies which connect the different organs. Thus, when the venereal impulse takes place in the corpora cavernosa of the penis, blood is solicited to the part, erection takes place, and the same afflux occurs in the vesiculæ seminales and vasa deferentia. When irritation commences in the latter from superabundance of semen, it occurs also in the penis; and in both cases the stomach and heart are influenced. As the functions of animal life might, perhaps, be brought to explain these circumstances, another example is chosen. When the stomach is in action during profound sleep or apoplexy, the tongue and eyes become red, the skin pale; afterwards the latter becomes hot and red, the circulation accelerated; the secretory organs of the alimentary canal are put in action; and this not merely from increased action of the heart, since muscular exercise does not produce the same effect. These facts show the influence of the ganglion system, not only on the muscles of the viscera, but also on the secretory tissues in general; and that this system is capable of inducing an afflux of blood to any part where it may be required, by its influence on the capillary vessels. There are many facts, also, which show that the nerves of animal life communicate to those of the viscera impulses which produce afflux of blood in their capillary vessels, alter their secretions, lessen the action of the ganglion system in some parts, and increase it in others; but they are not, in any instance, able totally to destroy its influence. Indeed, if the passions do, in certain cases, cause sudden death; it is by destroying the integrity of the brain, and abolishing respiration; disorders which appertain to the system of animal life. They may also abolish organic life by acting on the heart, rupture of which they sometimes produce; but they do not ever destroy the action of the capillary vessels so as to cause sudden death, without one of the preceding circumstances having been produced. It is thus that the ganglion system unceasingly protects organic life, without which the functions of animal life would soon become extinct. Many circumstances explanatory of the agency of the ganglion system in this respect are related by Dr. Lallemand in his Thesis, which we have so often had occasion to notice: these we need not mention here, as a full relation of them was given in our analysis of that work; and many curious facts and judicious reflections illustrative of these phenomena are adduced by Dr. Wilson, in his

Letters on Morbid Sympathies;* to which we must refer our readers, and terminate, for the present, the consideration of this interesting subject, as we have already extended it as far as the nature of our plan will permit.

Some curious (and, in the present state of our knowledge, apparently inexplicable) vital phenomena have lately been witnessed by Dr. Bostock,† in a patient who gradually lost the power of motion of the limbs, until at length it was totally abolished; the trunk of the body became also subsequently affected in a similar manner: whilst the senses and intellectual faculties remained unimpaired. As we shall detail the facts of this case in our "Critical Analysis," the remarks that might be made on them need only occupy us on the present occasion: but we feel equally unable with Dr. Bostock to offer any opinion at all satisfactory on the cause of phenomena so extraordinary, and involved in so much obscurity. Dr. Bostock observes, that it is remarkable that the voluntary power should be totally destroyed in all parts, while the other functions of the nervous system, as far as could be judged, were nearly unimpaired. The power of the nerves to convey impressions from their extremities was nearly in its natural state; while their power to transmit the acts of the will from the brain to the extremities was totally destroyed. The rigidity of the muscles, Dr. B. thinks, was not what ought to be denominated spasm, because there was no permanent contraction in them; and he is inclined to think it probable that it was an affection of the voluntary muscles, and not of the nervous system, in which the nerves transmitted their influence in the natural manner; while the muscles were incapable of receiving it, or of being acted upon.

We have to mention two instances of a phenomenon equally inexplicable with the preceding,—the change of colour of the secretion of the epidermis from black, or dark brown, to white. Although no useful application can at present be made of the knowledge of these facts, yet we feel obliged as historians, to notice them: they will, probably, be contemplated with more interest at some future period. One of the cases to which we allude occurred in the United

* *Practical Observations on the Action of Morbid Sympathies*, &c. Edin. 1818. See also *London Medical and Physical Journal*, vol. xl. p. 242 and seq.

† *Medico-Chirurgical Transactions*, vol. ix. part 1.

States, and has been related in this Journal;* the other was witnessed by Dr. Duncan, of Edinburgh.†

We refer our readers to the analysis of Dr. Lallemand's Thesis‡ for the detail of a fact now satisfactorily ascertained,—the communication of vessels in the united placentas of twin fœtuses; and likewise for some physiological observations respecting the formation and development of the placenta in cases of extra-uterine gestation.

We must also refer to a former part of our Journal§ for some interesting investigations respecting the power of distinct vision at different distances, by Mr. Littleton; as we cannot add any thing to the observations of that gentleman on this subject.

The *hybernation of animals* has long been an interesting subject for contemplation to philosophers; and physiologists have been much perplexed in their endeavours to account for the continuance of the life and warmth of the animal during the supposed cessation of respiration. Our readers, we presume, have been informed of the experiments some time since instituted by Professor Mangili, to ascertain whether or not there was a total cessation of respiration; and that he had been led to believe that the opinion generally entertained was incorrect: we need, therefore, only mention that, from a Memoir which he has lately published,‖ it appears that his former opinions were well founded,—that respiration is not entirely suspended, being merely extremely slow and inactive. A marmot in a dormant state, on being placed in an atmosphere of carbonic acid gas, becomes absolutely dead in the space of an hour.

Some new opinions on the pathology of apoplexy have been advanced by Dr. Abercrombie,¶ which bear the mark of original observation and judicious reflection that charac-

* See vol. xl. p. 391.

† *Edinburgh Clinical Reports*, p. 142. We shall relate the particular circumstances of this instance in the ensuing Number of this Journal.

‡ Vol. xl. p. 343, et seq.

§ Ibid. p. 177.

‖ *Dei Mammiferi soggetti a periodico Letargo*, dal Prof. G. Mangili. Pavia, 1818.

¶ *Edinburgh Medical and Surgical Journal*, vol. xiv. p. 553.

terize the productions of that author, and which are of considerable importance as regards the *practice of medicine*.

The cases we so frequently have occasion to witness, of persons being attacked with the usual symptoms of apoplexy and dying after having lain a considerable time in a state of coma, from which nothing can rouse them for an instant, when we cannot detect in the head the smallest deviation from the healthy structure,—have given rise to much hypothetical disquisition, but had never been satisfactorily explained. Indeed, terms and definitions have been advanced, instead of explanations: thus, a sthenic and asthenic state of the nerves,* and syncope,† have been adduced to account for the disease and cause of death, by men, whose reflections in other respects show much judgment and accuracy of observation. Our readers will recollect that, when treating on inflammation of the brain, Dr. Abercrombie expressed an opinion that the serum or other fluids effused in that disease could not be considered as the cause of coma and death, since they were so frequently found to have existed to a great extent where no symptom of compression of the brain had appeared. The development of this idea, and its application to apoplexy in particular, constitute the chief subject of the Memoir under consideration.

Dr. Abercrombie commences with remarking, that endeavours have been made to explain the cause of the species of apoplexy to which we have alluded, by the doctrine of increased determination of blood to the head; "but," he says, "I think it may be doubted whether this expression will bear examination, or whether it conveys any precise principle: the blood being propelled in every direction by an impulse primarily derived from the heart, it is not easy to conceive how, in the natural state of the parts, it should be propelled to the head with greater force, or in greater relative quantity, than to any other part of the body." We have always considered, that the doctrine which has been handed down to us from the days of Hippocrates—"*ubi stimulus, ibi affluxus*," had been one of the most firmly-established points in physiology. There are many facts on record which we do not conceive explicable on any other principle: such as a temporary cessation of pulsation in one radial artery, accompanied with coldness of the limb, while it continued natural in the other; and this occurring where *mechanical* vascular obstruction to the flow of blood cannot be supposed to have existed, and many other analogous occurrences. Although numerous instances of this kind might be adduced from different authors, we merely refer to the works of Dr. PARRY on Pathology and the Arterial Pulse *(passim)*; further testimony appears to us to be unnecessary. We think Dr. Abercrombie has erred in considering the circulation and distribution of blood as solely dependant on the action of the heart: this, however, is not a place for controversial discussion. We shall proceed to the analysis of the opinions of Dr. Abercrombie.

Dr. Abercrombie is disposed to consider that simple apoplexy depends merely on *interrupted* circulation of the blood in the brain; or such an obstruction to its course, that more blood enters by the arteries than can be freely returned by the veins. The causes of this interrupted circulation, he thinks, may be referred to the following heads:—

1. Derangement of the relation between the arteries and veins of the brain, in connexion with a general state of plethora. This position is illustrated by supposing an artery and a vein running side by side in a non-elastic canal, which they exactly fill; if, then, the quantity of blood usually sent to such a canal be increased, it is evident that the capacity of the vein must be relatively diminished, instead of augmented in a direct ratio: the consequence of this, it is evident, must be compression of the parts thus situated. This peculiar situation of the blood-vessels of the brain, Dr. A. considers, "lies at the root of the whole pathology of apoplexy. It is such as cannot occur in any other organ, because there is no other organ like the brain closely confined in a cavity of bone."

2. Causes which directly diminish the capacity of the venous system of the brain, or any part of it. When any considerable quantity of blood has been extravasated on the surface of the brain, coma is produced; and this is called compression of the brain. "But in what manner does this compression operate?" observes Dr. A. "We have no reason to suppose that the brain itself is capable of being compressed into a smaller compass, so as to make room for this extraneous mass. Something, however, must have yielded to make way for it; and this is most likely to be the vascular system of the brain. Less blood will, therefore, be now contained in the vessels of the brain, than was contained in them before the injury. If this diminution of quantity affected the arteries and veins equally, it probably would produce no urgent symptoms: but the quantity entering by the arteries is undiminished, or diminished only by the very trifling ratio which the quantity extravasated bears to the whole blood of the body; consequently, the compression will chiefly or entirely act upon the veins. From the smaller impetus of blood in them, they are less capable than the arteries of resisting its effects; and, from the situation of great part of them on the surface of the brain, they are more immediately exposed to it. Hence will arise a derangement of the circulation, analogous to that which I have supposed under the former article; more blood entering by the arteries than can be transmitted by the veins: the consequence is coma, or simple apoplexy.

3. Diseases of the sinuses, impeding the passage of the blood from the veins, or diminishing the area of the sinuses at particular parts.

4. Interruption of the circulation in the veins of the neck. The two latter classes of causes obviously act in the same manner as the second.

5. Diseases of the lungs and heart. Some cases in which a concurrence of these diseases with apoplexy was observed, are related; but Dr. A. does not adduce any reflections to explain their relation, or the mode in which they acted in the production of the latter disease.

We feel disposed to introduce here the opinion of M. Corvisart on this subject, as far as regards diseases of the heart. In the last edition of his work on Diseases of the Heart, he adduces the cases of Ramazzini and Malpighi, who appear to have died from apoplexy from this cause; and refers to Morgagni, Laurent, Lieutaud, and Testa, for examples: but he states that he has never himself witnessed death from apoplexy decisively caused by organic lesion of the heart. "I have frequently seen," he observes, "in cases of this nature, the whole cerebral vascular system, particularly the sinuses, gorged with blood; but I have never discovered extravasation of it in the substance of the brain, or in the ventricles. I have seen, also, in analogous affections, a collection of serum in the lateral ventricles, or at the basis of the cranium, and frequently an evident infiltration of it between the pia mater and tunica arachnoidea. And in the last hours, and sometimes during the last few days, of life, those patients are in a sub-apoplectic state. In many of these cases, death was somewhat sudden; but I cannot, however, assert that I have seen a single case in which apoplexy has been the evident effect of disease of the heart."*

The circulation in the brain may be interrupted by diminution of the impulse of blood entering the head,—as in syncope and disorders of extreme exhaustion. Dr. A.

---

* *Op. Cit.* p. 193.

enquires—"What is syncope, but an abolition of sense and motion? It is preceded by giddiness, tinnitus aurium, confusion of thought, loss of recollection, and failure of sight; and every surgeon has seen the syncope after blood-letting pass into violent convulsion. In what, then, does syncope differ in its symptoms from apoplexy?—only in the state of the general circulation; which in one is much weakened, or nearly suspended; and in the other is in full vigour." He also shows, that diminution of the quantity of blood will more affect the freedom of circulation in the brain than in other parts of the body: in the latter, the vessels will, to a certain degree, contract, and accommodate themselves to the lessened volume of blood; but the brain being closely covered from atmospheric pressure by the bones of the cranium, such contraction cannot take place so readily, and probably only in a very limited degree.

This, then, an impeded state of the circulation in the brain, is considered as the fundamental cause of apoplexy; and the author is disposed to doubt the correctness of serous effusion being one of its causes: it should be merely entitled one of the terminations of simple apoplexy.

"Did coma depend upon direct compression of the brain," Dr. Abercrombie observes, "we cannot conceive how six or eight ounces of fluid should exist in the cranium without producing it; but, upon the principle which I have proposed, of interrupted circulation, I think these cases may be accounted for. When such a quantity of fluid exists in the brain, the blood circulating there must be diminished by the same quantity; but, however much it may be diminished, while it continues to circulate without interruption, there will be no coma. I do not at present mean to investigate the manner in which this may take place, but the possibility of it is obvious. It may proceed from a general diminution of the mass of blood to such an extent, that the quantity sent to the head is lessened in proportion to the space occupied by the effused blood: and, even without this, we can conceive the possibility of the pressure within the cranium being so distributed as to affect the arteries and the veins equally."—"Pressure upon the surface of the brain I have supposed to produce coma, by diminishing the capacity of the veins, while the quantity of blood entering by the arteries remains undiminished: if both arteries and veins were affected equally, I imagine there would be no interruption, and no coma. These conjectures, I think, receive some probability from several cases of tumors, of great size, seated in the deep parts of the brain, which, though extensively affecting the organs of sense, have not produced symptoms of oppressed brain: while tumors of a smaller size, seated on the surface, have appeared to be connected with apoplectic symptoms."

Dr. Abercrombie has also published the commencement of some interesting reflections on coma, as it appears in a more chronic form: but we must defer the consideration of this subject until the full development of his views respecting it shall appear.

Some original opinions on this subject, which are supported by observation and argument, have also been advanced by Dr. Serres; but, for information on this point, we refer our readers to the last volume of our Journal.

Although, as we have already stated, the particular consideration of the second volume of the Transactions of the Association of the Dublin College of Physicians must be deferred to a future period, yet we are induced to embrace the following opinions on inflammation, in our history of the valuable physiological observations of the late period; although, perhaps, we are not doing strict justice to their author,* in thus isolating them from the series of reflections in which they are comprised.

"Inflammation, when it occurs, has its seat in those vessels, termed capillary, which form the medium of connexion between the termination of the arteries and the commencement of the veins; neither arterial nor venous, they form a separate system, exercise functions peculiar to themselves, and effect, by their agency on the blood, the various changes which are classed under the general head of secretion. When inflammation exists, congestion always takes place in the capillaries:—congestion in the capillaries is, in fact, a condition inseparable from the presence of inflammation, and may be traced to a disproportioned action between the capillaries and the minute arteries by which they are supplied. It is not necessary that the capillaries should be in a state of debility: it is sufficient that their action shall be less than that of the power by which the blood is impelled into them; for, from this cause, congestion equally follows, and inflammation must be the result.

"When congestion of the capillaries is accompanied by an increased action of the minute arteries, the *phlogistic diathesis* prevails; and the treatment consists in the employment of those means by which vascular excitement can be

---

* Dr. Grattan.

most readily subdued. When, however, congestion occurs in the capillaries without any obviously increased arterial action, the disease assumes the *chronic* form, and, if not relieved, pursues its course, exciting in appearance less disturbance, but proving in the end not less fatal, than the former. It is always in the capillaries that inflammation commences; for, when they exert themselves so as to transmit the blood as quickly as they receive it, the balance of the circulation is preserved, and neither congestion nor consequent inflammation is produced. If this were not the case, no organ could be considered secure against inflammation, under the slightest increase of the ordinary force and quickness of the general circulation: the most trifling exercise, or more than common exertion, must presently give rise to this serious disease. But, in general, when the force of the circulation is increased, the capillaries are equally excited to corresponding action, so that no accumulation of blood takes place. It is only when, from some local cause, their power of thus equalizing the circulation is suspended or destroyed, that inflammation is produced."

The natural organic and sympathetic connexion existing between the respective parts of the two lateral divisions of the human body, and the effects thence arising, have in all times engaged particular attention from curious medical observers; and many phenomena demonstrative of this connexion, and instances of occasional deviations from it, are dispersed throughout the works of numerous authors. Several medical dissertations* have been written on this subject; and it has been expressly treated, or casually adverted to, in a physiological point of view, by BORDEU,† LOSCHGE,‡

---

* DU PUI *Diss. Med. de Homine dextro et sinistro.* Lugduno Batav. 1780; recusa in SCHLEGEL *Thes. Pathol. Therap.* T. i. Nr. 1.—COURMETTE *Observations sur la Division de l'Homme en deux grandes Parties latérales,*—in Journal de Médecine, tome 85.—OSIANDER *Krankheitsgeschichte eines Mannes, der blos an seiner linken seite krank war, mit der Epikrise einer Einleitung in die Lehre von der Verschiedenheit der Krankheiten der r. u. l. seite überhaupt*; in *Abhandl. der Phys. Med. Soc. zu Erlangen,* B. i. 1810, p. 331.—MONTEGGIA *de Morbis Symmetricis et Asymmetricis*; in ipsius fasciculis Pathologicis, Mediol. 1789, Nr. i.

† *Recherches sur le Tissue Muqueux ou l'Organe Cellulaire.* 1769. p. 63 et seq.

‡ *De Sceleto Hominis Symmetrico. Præmittuntur quædam de totius Humani Corporis Symmetria.* Erlangen, 1795.

Isenflamm,* Bichat,† Heiland,‡ Meckel,§ and Ardieu.‖ It was lately proposed as a prize question by the Academy of Gottingen. Dr. Mehlis was the successful candidate on this occasion; he has treated the subject in his Essay in a perspicuous and judicious manner,¶ as it relates to morbid affections of the nervous system; gout and rheumatism; the circulation of the blood and action of the arteries; œdema and atrophia; diseases of the skin, glandular system, eyes, lungs, kidneys, and genital organs; herniæ; spinal distortion; several affections of the extremities; various anomalous symptoms, and metastasic phenomena; and the more general and evident differences in health and disease, between the two lateral divisions of the human body. Dr. Mehlis comprises in this treatise the most interesting facts that have been related by other authors; and introduces many original observations and judicious remarks, which will furnish much interesting matter for reflection to the physiologist, and tend to elucidate some obscurities in various diseases that embarrass the medical practitioner. We do not enter into the consideration of them, because our limits will not permit us to do it in a manner that would be at all useful to the student; but we considered that so interesting a subject should not be passed over without thus pointing it out to our readers.

Some very interesting discoveries, both in a MEDICAL and PATHOLOGICAL point of view, have lately been made respecting the nature of *small-pox*. It appears, from the observations of Dr. Thomson, Dr. Bent, Mr. Hennen, and Doctors Berard and Lavit, that an eruptive disease, partaking of the characters usually attributed to both *variola* and *varicella*, has been epidemic in some parts of France, England, and Scotland; and it is somewhat remarkable that

---

* *Uber der Verschiedenheiten d. r. u. l. seite;* in Isenflamm uber Rosenmüller *Beitr. f. d. Zergliederungskunst.* B. i. 1800. H. i. N. 2.

† *Recherches sur la Vie et la Mort,* art. iii. § i. and ii.

‡ *Darstellung der Verhältnisses zw. d. r. n. l. Hälfte der Menschl. Körpers.* Nürnberg, 1807.

§ *Beitr. z. vergl. Anat.* B. i. Leipzig, 1812; *und Handbuch der Menschl. Anat.* B. i. Halle, 1815.

‖ *Considérations Anatom. et Physiolog. sur la ligne Médiane, qui divise le Corps en Moitiés Symmétriques.* Strasburg, 1812.

¶ *Commentatio de Morbis Hominis dextri et sinistri.* Auctore Car. Frid. Ed. Mehlis, pp. 119. Gottingæ, et Londini, apud Treuttel et Würtz, 1818.

a similarity of opinions respecting the nature of those diseases should have been adopted by the gentlemen whose names we have mentioned from the contemplation of the facts which have occurred to their observation: a concurrence of sentiments which may obtain for them a greater degree of confidence than is generally conceded to opinions not confirmed by actual experiment, or supported by long-continued observation of similar series of phenomena.

This epidemic disease attacked three different classes of persons:—1st, those who had passed through small-pox; 2dly, those who had had cow-pox; and 3dly, those who had neither had small-pox nor cow-pox. The cases first observed at Edinburgh were of so mild a character, that the disease was considered as chicken-pox, till its severity and fatality in persons who had not undergone small-pox or cow-pox led to the opinion that, in all the different forms under which the eruption appeared, it could be no other than small-pox. This was rendered particularly evident by the disease that appeared in the castle in that city, which was transmitted by inoculation from a son of Mr. Hennen, who had had cow-pox ten years previously, and whose disorder was then considered by Dr. Thomson, as a well-marked case of chicken-pox. Mr. Hennen's son appeared to have caught the disease from a boy who had been vaccinated seven years before, and whose disorder also was considered as chicken-pox. This boy received the infection from a man who stated that he had had small-pox, but no mark of that disease could be found on him: the disorder with which he was affected was regarded as mild and distinct chicken-pox.

Six children, who had neither had small-pox, cow-pox, nor varicella, were inoculated with matter taken from Mr. Hennen's son. The disease produced was similar to that which has been termed modified small-pox, or like that complaint as it usually appears after vaccination. Several children, who had undergone either small-pox or cow-pox, caught a disorder from those children which assumed an equally mild character. Two adults, who nursed some of those children, and who had had variola, received from them a disease of considerable severity, which evinced the characteristic of modified small-pox,—a succession of crops of pustules during several days. One man who had neither undergone small-pox nor cow-pox, caught a disease showing all the characters of confluent small-pox; and of which he died.

Of seventy-two cases witnessed by Dr. Thomson, eight of the patients had passed through small-pox; twenty-seven, cow-pox; two had the disease co-existent with cow-pox; and thirty-five, including the six children which were inoculated with it, had neither had small-pox nor cow-pox. Three of the children affected with it after cow-pox had previously had an eruption of a similar kind; and in one of them, Dr. Thomson observes, he had the most satisfactory means of observing that it had each time exhibited the appearances which have been supposed to be characteristic of chicken-pox.

This epidemic disease usually commenced, observes Dr. Thomson, in a vesicular, or in a papular form speedily becoming vesicular, and became pustular only in some case during its progress. The pustules appeared sometimes with, and sometimes without, a central depression. The eruption was irregular in size and form, as well as in the place of its first appearance; and in almost all instances it came out in successive crops, some of which appeared on the body after the eruption was at the height on the face. It in general appeared,—even in severe cases, to have arrived at the height on the face by the sixth day of the eruption; and, in the milder, not unfrequently by the fourth or fifth day. The fluid contained in the vesicles and pustules, in a great number of instances, appeared to be lymph rather than pus, even to a late period of the disease, and generally dried into horny scales covering tubercular elevations of the skin, which, in several instances, were followed by pits or depressions of that texture. In the decline of the eruption, vesications upon an inflamed basis of a greater or less extent, frequently appeared upon the extremities, generally filled with lymph, but in a few instances with air; and, in some cases, small abscesses formed in the subcutaneous texture. This eruption rarely had any of the smell peculiar to small-pox. It produced but very little temporary blindness, and was seldom accompanied by the symptoms of secondary fever.*

In four of the eight persons who had undergone small-pox, this disease appeared in a highly aggravated and somewhat malignant form. Of the twenty-seven patients who had been vaccinated, not one died, and only three had it in a severe form. Of the twenty-nine who had it without having previously been affected with small-pox or cow-pox, nine died. In five of these fatal cases, the disease was of the sort described by Dr. ROGERS of Cork, and the late Dr. WALKER of Edinburgh, under the name of malignant crys-

---

* *Edinburgh Medical and Surgical Journal*, No. 56.

talline or water-pox. In the thirteen cases, although it did not prove fatal, yet it was more or less severe.

Although in other parts of the city of Edinburgh its progress could not be so accurately traced as its development in the castle, yet, in several situations, the mild and the malignant form appeared mutually to produce each other.

"The present epidemic," says Dr. Thomson, "appeared to me to resemble in every particular an epidemical eruptive disease which I saw prevailing extensively in the villages of Colinton, Slateford, and Currie, during the year 1809; and which, from a careful comparison of its symptoms in the milder cases with Dr. Willan's description of chicken-pox, I had concluded to be that disease. I was the more confirmed in my belief of these epidemics being chicken-pox, from my observing, at both periods, two symptoms occur in several patients, which have been regarded by Dr. Willan and others as diagnostic of chicken-pox. I allude to the succession in the crops of the eruptions, and the formation of vesications, of greater or less extent, resembling those made by scalding water, occurring among or in the interstices of the eruption, and producing the appearance which has been termed by some the *swine-pox*. I mention this circumstance with a view to show the reluctance and difficulty which I have had in adopting the following conclusions that have forced themselves upon my mind:—

"1st. I have been convinced, by the varieties which have appeared in the form of this epidemic in different individuals whom it has attached, that the descriptions which have been given of the appearances and progress of the eruption in small-pox by our best systematic authors, are, in many respects, imperfect; that the diagnostic marks which have been pointed out between small-pox and the disease that has been termed chicken-pox, are not to be relied upon; and that no applicable marks of distinction between modified small-pox and chicken-pox have hitherto been established.

"2dly. It appears, from the records of medicine, that the same person may have small-pox twice (if not oftener) during life. It is an event which I conceive must have occurred frequently, though its occurrence is denied by some, and comparatively but few instances of it are upon record, even by those who believed its possibility.

"3dly. It has been, I conceive, incontrovertibly established by Dr. Jenner and his followers, that cow-pox has the property of rendering those who have passed through it much less susceptible of small-pox infection than they were before; and, besides this, that it possesses also the invaluable property of modifying the small-pox in those who receive them, and of converting them, from the most fatal of all diseases, to one scarcely, if at all, fatal.

"4thly. By admitting that small-pox possesses this modifying property, it will follow, that, in the instances in which they excited this previously to the discovery of cow-pox, they must have produced a mild and less fatal species of small-pox, but a species which has not been recognized or pointed out as differing from primary natural small-pox by any author with whose writings I am acquainted. It seems, therefore, probable that this secondary small-pox, which we have now so much reason to believe was of frequent occurrence, must have formed a considerable portion of the varioloid eruptions that were formerly denominated the spurious small-pox, and afterwards by some the chicken-pox.

"5thly. After Dr. Heberden had distinguished chicken-pox from small-pox, and had convinced himself and the medical world that these diseases arise from two contagious poisons, specifically distinct from each other, it seems probable that the cases of modified secondary small-pox, which may have occurred, must have been described as cases of chicken-pox.

"6thly. I am satisfied that, previously to the discovery of the cow-pox, secondary small-pox, being a disease frequent in its occurrence, must have stood in nearly the same relation to primary small-pox that modified small-pox now stands in to cow-pox; and my present impression is, that it may be that chicken-pox and modified small-pox are one and the same disease.

"I am not aware of any accurate or extensive series of observations which contradict this hypothesis; nor do I think that it can be well set aside, till it shall be proved that chicken-pox occur generally in persons who have not passed through cow-pox or small-pox, and prevail epidemically without cases of small-pox appearing among them. There are upon record, it is true, many cases in which the spurious or chicken-pox are said to have intervened between the cow-pox and the modified small-pox. Before, however, admitting that, in the production of these cases, there operated two poisons distinctly different, it will be necessary to be assured that the appearances exhibited by chicken-pox cannot be produced by the contagion of primary small-pox, and *vice versâ*, as well as that the contagion of small-pox cannot produce an eruptive disease twice in those who have undergone cow-pox inoculation. It will be necessary also to ascertain whether those who have passed through small-pox in its milder form are equally secure against a second attack of small-pox, as those who have passed through the disease in its more regular and severe form.

"7thly. It seems to me certain, that the epidemical disease, which has of late prevailed in Edinburgh, is the same with those varioloid diseases which, since the introduction of cow-pox inoculation, has been observed in many places of this and other countries; and which have been, by some medical practitioners, regarded as small-pox, and by others as chicken-pox.

"Lastly.—It seems to me, that the hypothesis which I have thrown out, if it shall be confirmed by future experience, will afford a satisfactory explanation of the nature of those varioloid diseases which have of late years been observed to succeed to the practice of cow-pox inoculation; and will, at the same time, reconcile the various and discordant opinions which have been entertained by medical practitioners respecting these diseases.*

The notions respecting this disease which Mr. Hennen has been led to form, may be understood from the following queries which he has proposed respecting this subject:

"Do variola and varicella, when they happen to be contemporary diseases, modify each other?

"When thus modified, are they capable of producing an anomalous disease, in the same way as a disease of that description was produced by Doctors Woodville and George Pearson, by vaccinating at the Small-pox Hospital in London?

"If they do not modify each other, but remain distinct unmixed diseases, will one of them (variola for instance) attack one set of individuals in the same town, house, or family, while varicella attacks another set?

"Does varicella ever occur epidemically without small-pox; and where are the records of such epidemics to be found?

"Is there, in the epidemic that has lately appeared in Edinburgh, any peculiarity, either in the mode of attack, progress, or decline, which authorize us to call it a disease *sui generis?*

"Lastly. Have the eruptive diseases above mentioned any, and what, connexion with the reigning epidemic fever?"†

For an account of the facts observed by Dr. Bent, we refer our readers to the last Number of our Journal: we shall in this place detail his reflections on them, in order that

* *Op. cit.* p. 522, and sequent.

† *Op. cit.* p. 464.

the principal opinions that have been formed on this subject may be contemplated at one view.

"The appearances which have been presented in different individuals," Dr. Bent observes, "have been extensively varied; and I can venture to affirm, that every gradation imaginable, between the mildest form of chicken-pox and a severe small-pox, has been observed. If I were to make any general conclusion on this part of the subject, it would be, that, in the majority of examples, the disease has exhibited those particular appearances which Dr. Willan has described as occurring in the instances where the influence of cow-pox has modified small-pox:—'The pocks have a vesicular character, and often a small horny appearance, and pass through the usual appearances; but, instead of proceeding to full suppuration, they begin to subside, and dry away on the sixth day from the commencement of the eruption.' A very curious and important feature in the history of this disease is, that *the general symptoms and the particular character of the eruption are the same in those persons who have never had the cow-pox as in those who have passed through that disease satisfactorily*."

"With varicella, the disease of which I am treating has many symptoms in common, and numerous examples appear identical with it; but, in very many others, it is more severe, of longer duration, and more fatal in its consequences, than I have ever witnessed or seen described in that complaint.

"The great fickleness and inconstancy which the disease presents have led me to consider, whether modern physicians have not erred in making variola and varicella distinct diseases; and whether Morton and Sydenham, and their contemporaries, who considered them as varieties merely of the same disorder, may not prove to be the more accurate observers. The disease has proved fatal in many instances amongst the lower orders, who unfortunately retain the old prejudice respecting the necessity of encouraging eruption by means of flannel and fermented liquors. Under better treatment, it has also occasioned some deaths. One in-patient at the Infirmary, who had previously had the vaccine disease; and two out-patients, one of whom had also had it, have been victims to it.

"May not the contagion of small-pox, after having been introduced into the system of an individual, or a succession of individuals, who are under the protection of vaccine disease, have its qualities so changed as to allow of its propagating a disease in others, free from vaccine influence, similar to that by which it was itself produced? If this conjecture should prove well-founded, the prevailing disease may be deemed a new variety of small-pox.

"The conclusions which I am inclined to make from the facts which have hitherto been presented to me, are—

"1st. That the disease which I have described is not the genuine small-pox.

"2ndly. That it differs from chicken-pox in duration, severity, and consequences.

"3dly. That it is not merely small-pox modified by vaccine influence, because the symptoms are the same in those who have not had vaccine disease as in those who have.

"4thly. That vaccination has an imperfect, if any, influence in arresting its attack."

It appears from a work recently published by Doctors Berard and Lavit,* that a disease, showing all the characters of that we have just described, was epidemic at Montpellier at a late period. The history of the disease is, indeed, so precisely similar, that it is quite unnecessary to enter into the particular consideration of it. The anomalies and varieties of its appearance induced these gentlemen to believe that they could, in different cases, distinguish both the eruptions of variola and varicella.

"To what exanthematous disease other than varicella," they observe, "can we refer pustules, which, on the fourth, third, second, and even the first, day of their appearance, rise with such rapidity that the eye can almost distinguish their progress from hour to hour, becoming filled with serum, at first limpid, afterwards opaque, white, and thick, without ever showing the characters of true pus; pustules which bursting at length become dry, or empty themselves, leaving superficial scabs, which fall off in scales or harden into tubercles?

"On the other hand, to what other disease than legitimate variola can a great number of the traits of the disease which we have described be attributed? Is not the eruptive fever which is proper to that malady evident in the following symptoms:—nausea, fainting, somnolence, stupor, pain in the loins, oppression, continuance of the primary fever during three or four days, and its disappearance immediately after the eruption? Do we not recognize it in the progress of the disease in general, and that of the appearance of the pustules, which commenced most generally in the face, and

* *Essai sur les Anomalies de la Variole et de la Varicelle, avec l'Histoire Analytique de l'Epidémic éruptive, qui a régné à Montpellier en* 1816; par M. F. Bérard, M.D. &c. et M. Lavit, M.D. &c.

terminated on the extremities? The peculiar form of the variolous pustule, in the depression in the centre, with a dark spot in the middle of this depression, and the inflammatory areola with which they were surrounded? The suppurative fever, which, when it was accurately attended to, took place in a great number of instances on the ninth, the eleventh, or the twelfth day,—do they not characterize variola? Lastly, the duration of the disease in these cases was not less than twelve days from the primary fever to the desiccation of the pustules."

Such were the characters of the disease which formed its boundaries, but there were between these many anomalies witnessed; such as we mentioned in the epidemic which appeared in Edinburgh and Derby.

After many reflections of the authors, which we shall not adduce for the reasons assigned for not entering into a full consideration of their observations, they say, "Is not varicella an imperfect, altered, or modified, species of variola?"

We shall conclude this subject with the following observations of Dr. Monro on small-pox, as it affects those who had previously undergone that disease:—

"I have lately had occasion to meet with four persons who have had small-pox twice in the progress of life, and three of these were very much marked by the first attack of the disease.

"The second attack of the small-pox is on some occasions mild, but in other instances malignant and fatal; and of both varieties I have lately had occasion to see examples.

"In the milder, the pimples pass at once from the state of vesicle to that of incrustation: therefore the disease has been called *horn-pock*, on account of the smooth horny appearance of the crust elevated on a hard warty-looking base.

"This form of small-pox is of a mild nature; is attended with no secondary fever, though the crusts do not fall off so soon as in the casual small-pox, when the contents of the pustule become purulent. This appearance has been observed by many persons, in describing this eruptive disease after vaccination, which seems to afford proof of an analogy between the effects of small-pox and cow-pox on the constitution. There is, however, this essential difference, that the small-pox which succeeds the former has sometimes proved fatal.

"It is a singular fact with regard to the contagion of small-pox, that, though the system be unsusceptible of the contagion when it is floating in the air, yet the disease may occasionally be communicated even to those who previously have had the small-pox by inoculation: hence several surgeons who have practised inoculation for years, by unguardedly pricking their fingers with the point of a lancet charged with variolous matter, have got the disease for a second time; and in this manner students, in dissecting bodies of persons who have died from small-pox, have received the contagion of that disorder for a second time."*

Several valuable works have been published on *Epidemic Fever*, as it has lately prevailed in different parts of the United Kingdom; the most important of which are those of Doctors YULE, GRAHAM, MILLER, BROWN, ROGAN, KIDD, Mr. BONNAR, and especially that of Dr. BATEMAN, and the *Clinical Reports* of Dr. DUNCAN, jun. It does not come within the scope of our plan to enter into the particular consideration of these works, since they chiefly tend to develop the views respecting the nature of that disease, and confirm the propriety of the mode of treatment which have been for some time past adopted, and which have been so fully treated of in different parts of our Journal. We shall, however, adduce the general observations of Dr. Bateman and Dr. Duncan, which will show the opinions respecting it that must be considered of the most weight, and are most generally acknowledged at the present time.

"All the attention which I have been able to give, during fourteen years, to the passing phenomena of fever," says Dr. Bateman, "and more especially the observations which I have made while several hundred cases have been presented to my view within the compass of a few months, have tended more and more to impress me with the conviction of the identity of that disease under all its modifications. Its character, indeed, is greatly varied by the different circumstances in which it occurs: by the age, constitution, and previous health, of the patient; by the intensity of the exciting causes; by the situation and season; and by early neglect or mismanagement: but it is not more varied than other febrile diseases,—the small-pox, for instance, or scarlet fever,—under similar circumstances; and examples of the most distinct modifications which it undergoes are often observed in individuals of the same family. Thus, in the instance of a man and his wife, who were brought to the House of Recovery together, the former was affected with the mildest symptoms of fever, which scarcely confined him

* *Observations on the different kinds of Small-pox, and especially on that which sometimes follows Vaccination*; by Alexander Monro, M.D. F.R.S.E. &c. Edin. 1818.

to bed, and terminated in a speedy convalescence; while his wife was lying in a state of stupor, her skin covered with *petechiæ* and *vibices*,—in a word, exhibiting the most formidable symptoms of the worst form of typhus. Yet these extreme degrees of the disease manifestly originated from the same cause; and it would be equally unphilosophical to account them different kinds of fever, and give them distinct generic appellations, as in the case of the benign and confluent small-pox, which are generated in like manner from one contagion.

"The older writers, under the influence of system and hypothesis, greatly erred in the multiplication of the species and appellations of fever; and Dr. Cullen performed a material service to pathology, when he reduced the multitude of names to three genera, under which they were ranked as symptoms. But the propriety of the three genera which that able nosologist established, is very questionable. With respect to the first, *Synocha*, his distinguished successor in the professional chair, above alluded to, Dr. James Gregory, asserted that, during thirty years' practice, he had never seen a purely inflammatory fever unconnected with acute inflammation of some organ; and my own subsequent experience entirely coincides with that assertion. It cannot be doubted, as Dr. Gregory remarked, that the *causus*, or ardent fever of the ancients, was the endemical bilious remittent of hot climates; and that no continued fever of this country assumes that character. This genus, therefore, should be discarded, and in fact occupies no place in the treatises on fever which have lately appeared.

"In constituting the genus typhus, which, it is evident from the long list of synonyms, comprehends the principal varieties of continued fever, Dr. Cullen found that his definition excluded some of the instances in which the heat of the skin was augmented, and considerable vigour of pus e occurred, especially at the commencement; and he deemed it necessary to establish another genus, which he arbitrarily called synochus. But he candidly questions the propriety of this classification, and acknowledges that there is no essential distinction, no line of separation, to be drawn between the two; the synochus being in fact typhus, only somewhat more inflammatory in the beginning. And he thus appears to have fallen into a dilemma, by an attempt to institute an artificial distinction, which was inconsistent with the phenomena of the disease. It would have been more consonant with these phenomena, as well as with the principles of classification adopted by natural historians, to have made fever a genus, and marked its varieties; or rather, as typhus has become the popular appellation of the fever of this country, to have extended the definition so as to include, as Dr. Armstrong has done, the inflammatory as well as other varieties of the disease."*

"I have not used Cullen's distinction of synochus and typhus," observes Dr. Duncan, "because I do not believe that the distinction exists in nature. I have never seen an instance of typhous fever according to his definition. All our severe fevers begin with excitement and terminate in debility, or are instances of synochus; although, in truth, they are the identical disease from which Cullen drew his description of typhus, and are genuine examples of the only typhus fever which exists.

"I have preferred distinguishing the cases by the epithets cephalic, pulmonic, gastric, enteric, hepatic, &c. from the principal organs affected; for, although frequently the functions of all were somewhat disturbed, the force of the disease seemed generally to bear upon one or two, and sometimes upon different organs in succession."

While the preceding observations were being arranged for the press, the second volume of the Transactions of the Association of the Fellows and Licentiates of the College of Physicians in Dublin came to our hands: we have just time to make some abstracts from the reports of the physicians of the Fever Hospitals of that city, which will serve, with those we have adduced from the works of Drs. Bateman and Duncan, to complete the account of the opinions held throughout the United Kingdom on the points of most importance respecting the origin and character of the reigning epidemic.

"If proofs were wanting," observes Dr. Stoker, "that deficiency as well as vitiation of nutriment is a chief source of epidemics, these would be abundantly supplied from the history of the present fever. The dearth produced by the failure or injury of the crops of 1816 was quickly felt, and was soon accompanied by the usual attendants of famine amongst the lower orders of this country, whose poverty was before sufficiently deplorable: accordingly, fever first appeared in those parts of the country where famine most prevailed, and every where succeeded it in the same degree. In many instances, of which Dublin presented a prominent example, the supplies of wholesome nutriment to the poor, by the humanity and munificence of the affluent, were found not only to prevent a greater prevalence of fever than usual

* *A succinct Account of the Contagious Fever of this Country*; by Thomas Bateman, M.D. F.L.S.

for a considerable time after the inhabitants of other parts had suffered severely from it, but also to check its progress sensibly after it had appeared.

"It is, I think, difficult to say at what time it began to be propagated by contagion, or to what extent; as the same scarcity of provision in which the fever originated was found ultimately amongst all the poorer classes in Ireland, and was aided by the strong predisposition given by depressing passions; and probably, according to the language of Sydenham, 'a certain constitution of the atmosphere,'—a cause of pestilence known to the ancients, and described in the natural but affecting words of Virgil:—

'Subito cum tabida membris,
Corrupto cœli tractu miserandaque venit,
Arboribusque satisque lues et lethifer annus,
Linquebant dulces animas aut ægra trahebant
Corpora.'

"Where the chief cause,—namely, deficiency of wholesome nutriment,—did not exist, the epidemic was not in general found to spread: thus there were many families of condition, in each of which I have attended an individual in the worst forms of this fever, but cannot call to my recollection any instance in which it has communicated to other members of these families.

"The symptoms of this epidemic resemble very nearly those of the fever which has been so long known in this city, and may be classed under the heads of typhus mitior and typhus gravior, mingling with others either constitutional or incidental: the mild forms bear a very large proportion to the more severe, and the usual course is from three to nine days; most generally terminating favourably on or before the seventh.

"Using the appellations typhus gravior and mitior to denote different degrees of the same disease, I shall, in my general descriptions, sum up the symptoms of both.*

"The scarcity of provisions last year," Dr. O'Brien remarks, "combined with the consequences of the sudden termination of the war, had increased the misery of the poor to its utmost height; and from this period we may date the origin of the present epidemic fever which has made, and continues to make, great ravages through the kingdom. These evils, operating in a wider sphere on the dense and crowded population of this and the other great cities, have produced more fatal effects, as might be expected, than

* *Medical Report of the Fever Hospital and House of Recovery, Cork-street, Dublin.*

elsewhere, and appear to verify the observation of Mr. Malthus, that pestilential diseases are the inevitable attendants on a population too great for its means of subsistence.

"From the vast variety to be observed in fevers, both as to duration and intensity, their classification is extremely difficult, if not impossible. The division of Dr. Cullen is palpably indistinct, according, indeed, to his own confession; and his two genera, typhus and synochus, graduate into each other with such minute shades of difference, that physicians, the most familiar with their habits, will sometimes differ in denominating them. Hence a great irregularity appears in our previous annual lists: one year, the quantity of typhus appears too small, on another enormously large; whilst there is little difference in the total amount of fever. I have thought it better, therefore, to omit here the division altogether, and retain only the name of the natural order.

"Of the contagious nature of the fevers of our climate, some doubts have been lately expressed, chiefly, I believe, by some medical gentlemen of the army, who had long been accustomed to the remittent fevers of warm climates. On this subject an interesting pamphlet has been lately published by Dr. Stokes, late professor of physic in our university, who appears to me to have refuted the opinion in a very satisfactory manner. Although these opinions have been very little attended to by the public, and further argument is unnecessary on a subject which public opinion has long since decided; yet I cannot help mentioning, in illustration of this subject, a few facts, which have fallen under my own immediate observation, in relation to the medical officers and attendants at the Fever Hospital, Cork-street. Of nine physicians, who have acted as permanent physicians to the Fever Hospital since its foundation, five have had contagious fever, and two of the five have died of that disease: of the four remaining, two had contagious fever previous to their connexion with the hospital. Of eight apothecaries, only one escaped. Of the nurses employed in the hospital, only one individual, as far as I can learn, who has resided for a year or upwards, has had contagious fever; and some have had the disease three or four times.

"The escape of so great a proportion of physicians, who must every day handle and minutely examine between forty and fifty fever patients, appears to prove that the disease, though certainly contagious, is not very contagious; and it is only probably under circumstances of strict and frequent intercourse with the sick, when frequent contact takes place, or the patient's breath is inhaled by the organs of respiration, that contagion is capable of producing its effects in our climate."*

The space this subject has already occupied of limits too restricted for the numerous objects they must comprise, obliges us to be very concise in our abstract from the report of Dr. Barker: we can merely at present notice his opinions respecting the origin and mode of dissemination of the epidemic, and his general remarks on its symptoms; his learned and very judicious illustrations of this subject, as well as the reports of Drs. Stoker and O'Brien, shall engage our particular attention at a future period.

"The causes which served to diffuse this fever, and render it epidemic, may be divided into two classes—*immediate* and *concurrent*.

"Its chief *immediate* cause, in my opinion, has been *contagion*; and to this its origin and diffusion are principally or solely to be attributed.

"Among its *concurrent* causes, I would place first the miserable condition of the lower class at this period. Famine, however, operates *indirectly*, and as a concurrent cause only, by promoting the spreading of the contagion, and rendering the human system more liable to receive it. By increasing their poverty, it obliges the poor to crowd within narrow limits in small lodgings; and, by bringing a number of persons within the infected circle, it augments the risk of infection, and diffuses contagion. Poverty, aggravated by famine, increases the susceptibility of fever, by causing despondency: persons reduced in their circumstances have been frequently observed to suffer from fever. Among the concurrent causes, the failure of fuel also deserves notice:—hence arose the crowding of apartments, in order to obtain warmth; and diminished ventilation, since a fire in the flue must constantly renew the air; and with these consequences of the want of fuel must have been combined an increased difficulty of cleansing either wearing apparel or dwellings.

"As the causes of this fever did not differ materially, except in degree, from those which are in continual action in this city, so the symptoms did not afford any great deviation from the usual course. Cases, however, presenting a discordancy in their symptoms were more frequent than usual, constituting the *fievre ataxique* of French authors. With the more frequent occurrence than usual of irregularity in the symptoms of fever, the frequency of petechial erup-

* *Report of the Sick-Poor Institution*, Dublin, 1817.

tions was also remarkable: these were exemplified in persons of all ages; and that peculiar form of petechiæ in which the eruption resembles the measles, occurred much oftener than heretofore. Another peculiarity in this epidemic, at its commencement frequently, and during the winter season, was the occurrence of a livid or purple colour of the extremities, sometimes of the feet only, but generally of the hands also, not accompanied by coldness of these parts. It was mostly attended by great disturbance of the sensorial functions. It was a formidable, though not a mortal, symptom, as it was formerly observed to be. This symptom was observed in severe cases only, which constituted a small proportion of the whole number of patients."*

Although it would appear that difference of opinion on the nature of the *bilious remittent fever of hot climates*, or as it is commonly termed, *yellow fever*, could not exist after attentive and unprejudiced consideration of the facts that have been made public; yet we find that indecisive notions respecting it still prevail. Were this observed only in a few individuals, it would not engage our attention; but when we find a body of the medical faculty, certainly not inferior in learning and talents to any in Europe, lately express doubts respecting the contagious nature of that disease, it leads us to embrace with eagerness the consideration of facts recently witnessed, which appear to decide this question in the most incontrovertible manner. We allude to those related by Dr. Musgrave of Antigua, and which are published in the ninth volume of the Medico-Chirurgical Transactions. We shall at present merely adduce the general conclusions that may be drawn from the observations of Dr. Musgrave: the particular facts on which they are founded will be detailed when we enter into the consideration of the work in our "Critical Analysis."

The local peculiarities of the country under which yellow fever appears, are—a marshy soil, with a certain height of temperature of the atmosphere; and it has been observed, that its prevalence is much increased after a considerable fall of rain. *Excessive* heat was not found to be particularly favourable to its production.

The subjects of it were principally strangers, not merely Europeans, but persons lately arrived from the neighbouring islands. Among the natives, those of a debilitated habit of body, and who had been confined to a poor diet, were

* *Medical Report of the Fever Hospital*, Cork-street, Dublin, 1817 and 1818.

most frequently attacked, and suffered most severely from it. Such a distinction could not be well ascertained amongst Europeans, either in liability to the disease or the degree of its violence. No instance of its occurrence in any but a white subject was observed. The inhabitants, both white and coloured, who did not suffer from fever in *some form*, appeared to be more than usually exempt from other diseases during the period at which this epidemic reigned.

Not a single instance was witnessed which could not be accounted for in a manner the most perfectly satisfactory, without attributing to contagion the most trifling agency.

The fever affected persons twice, in several instances, and was occasionally fatal in the second attack. The diagnosis of this disease, proposed by Mr. Pym,—the existence of the *black vomit*,—cannot be considered as applicable to it in general; as no instance occurred of the recovery of any person who had experienced that symptom.

It sometimes commenced as simple intermittent fever; and some of those cases terminated fatally, after having displayed all the symptoms most decidedly characteristic of yellow fever. Several cases were also observed, which commenced with the usual symptoms of yellow fever, and afterwards assumed an intermittent type.

Intermittent fever was at the same time unusually prevalent and severe, particularly amongst the black inhabitants of the island; although, as before observed, no instance was witnessed of their being affected with that form of disease constituting what is usually considered as yellow fever.

The above facts seem to prove, that yellow fever arises from marsh miasmata, and is essentially identical with remittent fever of other countries; only modified by the influence of climate, habit of body, &c.

When treating on Pathology, we mentioned that Dr. Bricheteau had witnessed some cases of apoplexy from peculiar softening of the brain: we shall now detail the symptoms which usually appear in the different stages of this disease. We feel it necessary to remark, that the author speaks with much indecision, from the want of satisfactory information on this subject, in consequence of the varieties of character which they have assumed in different instances.

A remarkable diminution of the muscular power is observed in the first instance, and the patient frequently complains of sudden loss of command over the motions of the limbs; many stagger in walking, as if they were drunk. A progressive weakening of the mental faculties is also observed, almost constantly accompanied with continual cephalalgia, more or less violent in degree. Sometimes incomplete hemiplegia takes place; the appetite remaining good, and nutrition well performed. After a greater or less length of continuance of this state of derangement of the health, and of suffering to a greater or less degree, symptoms of compression of the brain appear, assuming the character of an ordinary attack of apoplexy; and the patient universally dies in a few days, and not unfrequently in a few hours.

Although a series of forms of disease may be essentially similar in their nature, the variation observed depending only on the degree of its intensity or some accidental circumstances, and, therefore, require the same general measures for their relief; still it is of great importance, in many instances, that those different forms should be accurately defined, particularly as regards the greater or less rapidity of their progress; for these circumstances, and especially the latter, must have considerable influence on the mode in which the measures for their relief must be employed. On this account, the Memoir of Dr. ABERCROMBIE on *Inflammation of the Brain* is highly valuable: we need not, however, enter into the consideration of it at present, since it engaged our attention on a former occasion.*

As every thing relative to *hydrophobia* must be considered of considerable importance, we shall detail the principal facts related in a Memoir presented to the Medical Society of Paris, by M. MORELOT; although we feel it necessary previously to observe, that the existence of rabies in the animal, the actions of which gave rise to the following observations, is by no means satisfactorily proved.

A wolf, on entering a village (September 17), was actively pursued by a dog, when he encountered a boy, John Jabœuf, aged 13 years, fell on him, bit him in several places about the head and left hand, and would probably have devoured him if the dog had not prevented it. Twenty-four hours after the accident, the wounds were washed, and cauterized with the muriate of antimony. On the following day they were dressed with an escharotic digestive. The use of a drink made warm with volatile alkali, was ordered for him. The boy was much terrified at the time of the accident, and from that period his imagination was much affected. Fourteen days afterwards, the usual symptoms of hydrophobia appeared, and on the seventeenth day he died. On opening the body, the membranes of the brain were found covered

* See vol. xl. p. 315.

with coagulable lymph, and its vessels gorged with blood. The salivary glands, fauces, pharynx, larynx, trachea, and alimentary canal, as far downwards as to the ileum, showed signs of inflammation; and the stomach was filled with a yellow fluid.

The wolf was pursued by the dog for some time after the above incident, until he at length escaped from him. On the following day, he appeared at a village about a league and a half distant from the former. Claudius Sauvageot, a boy 13 years of age, was his first victim: he was bitten on the head, neck, and hand. The same treatment as that employed for J. Jabœuf was adopted, (the period that elapsed previously to its application is not mentioned.) On the seventieth day from the accident, the boy lost his appetite; an abundant flow of saliva took place; and, without having shown any particular aversion to liquids, he died in the evening, without having suffered any violent emotion; having preserved his natural sprightliness of disposition to the last.

Jane Joliot, aged 37 years, was the second person attacked by the animal on that day: she was bitten on the lip, gums, nose, and forehead. The same treatment was adopted, (period after the accident unnoticed); and she left the hospital forty days afterwards, apparently well: but Dr. M. was informed that she died of hydrophobia some time afterwards, after having suffered from it for twenty-four hours.

M. Robiquet, aged 40, the third person attacked, was bitten on the thigh: the same treatment was employed, and she has not experienced any ill consequences.

F. Gerbeau, aged 37, in the ninth month of pregnancy, received several wounds on the face and neck immediately after M. Robiquet: the wound in the neck laid bare the trachea to the extent of an inch. The same measures were adopted, and with equally fortunate results.

C. Brugnot, aged 24, was afterwards encountered, and bitten on the forehead and nose. The same results ensued from a similar mode of treatment.

Lastly, Stephen Maniere, aged 27, put a stop to these accidents by strangling the animal in his arms: this was not effected without the reception of several wounds,—but they were on parts covered by his clothes. The results in this case were equally fortunate.

The wolf was dissected; the only circumstances noticed were—a dryness of the pharynx, œsophagus, and larynx. The stomach was a little contracted, and contained some filaments of dry vegetables. The thoracic and abdominal viscera did not show any morbid appearances. It is not mentioned whether or not the brain and spinal marrow were examined.

In the cases above mentioned, the symptoms of hydrophobia are only satisfactorily ascertained to have appeared in John Jabœuf: and, from the want of other facts to substantiate the existence of rabies in the animal, many persons will doubtless be inclined to attribute the disorder of that boy to the influence of the imagination. The terror arising from the attack of such an animal, and the affection of mind under which he subsequently laboured, might, not unreasonably, be supposed sufficient to produce the *inflammation of the brain*, and other symptoms that appeared.

The account related of the second case is not such as to lead us to believe that the patient died of hydrophobia. Neither the characteristic affection,—the disturbed state of mind,—the convulsive actions,—nor, indeed, one symptom designative of that disease are noticed to have appeared: on the contrary, it is said that the boy preserved his cheerfulness to the last, and died *tranquilly (il à succombé tranquillement)* after an illness of a few hours.

With respect to the third case, Dr. Morelot merely heard from common report that the patient had died from hydrophobia. The disposition of the vulgar to consider every febrile disease, particularly if accompanied with mental disturbance, as that disease, after similar accidents, is well known: their interpretation of it does not merit serious attention when other facts do not tend to support it.

If, however, the existence of rabies in the animal be admitted, the results of the prophylactic measures that were adopted must be considered very interesting. The measures employed were certainly judicious. Many facts indicate the benefit that may be expected from preserving a discharge from the wound for a considerable period. It has also been remarked in the few cases which have terminated favourably, that copious sweating has generally appeared. Such an effect might be expected from the use of volatile alkali; which might also prove beneficial from the same reasons that it is in cases of poison from venomous serpents.

There are but few occasions on which it can be considered that an advantage would ensue from comprising various morbid affections of different organs under one general term; but that beneficial effects may thence arise in some instances is highly probable, and such a consideration has induced Dr. Marshall Hall to include under that of mimoses the affections usually denominated *dyspeptic*, *hypochondriac*,

*bilious*, *nervous*, *chlorotic*, *hysteric*, *spasmodic*, &c.;* from such an appellation pointing out the imitative, multiform, various, and changeable character of their symptoms; and tending to avert the evil that has arisen from describing those diseases by terms conveying, in most instances, erroneous notions of their nature; at the same time that the practitioner, incited by the ideas it conveys, may, perhaps, thence be led to a more cautious and accurate mode of investigation. The obscurity in which these affections is involved, in consequence of the absence of particular local uneasiness; and from the symptoms, as expressed in the words of Hippocrates taken as the motto to this work by Dr. Hall, being for the most part not peculiar to any one of those diseases, many of them attending several, and some of them all,—renders a general term of this kind in some degree advisable in the present state of our knowledge. Our intention at present is merely to notice the general views of the author on this subject; the particular consideration of them being better adapted for another occasion.

The disorders of the chylopoïetic viscera noticed in this work are described under the terms MIMOSIS *acuta*, *chronica*, *decolor*, *urgens*, and *inquieta*. The first species is usually accompanied with head-ach, vertigo, stupor; cough, viscid expectoration; paroxysms of oppressive dyspnœa; palpitation of the heart, frequency and irregularity of the pulse; frequent and violent hiccough, vomiting of food; some convulsive and spasmodic affections; pain in the epigastric, or one or both of the hypochondriac or chondiliac regions; constipation, diarrhœa, tenesmus; melœna; icterus; severe pains of some of the limbs; and considerable emaciation of the body. The disorder of the chylopoïetic viscera, although at first merely functional, often terminates in organic disease; and the same consequence also takes place in the parts which are secondarily affected,—as the heart, lungs, and brain. "In some cases, the disease degenerates into a state resembling *cachexia*, and has been accompanied with other morbid affections,—especially of the skin, the mouth and throat, the periosteum, the absorbent glands, &c. Some of these have resembled syphilitic affections; others have exhibited very different appearances." The mimosis chronica is denoted, in general, by fits of despondency and gloom, of invincible disinclination for exertion, of pain about the head, sinking at the precordia, and heat or fulness of the stomach. This form is intimately allied to the less severe and more continued form of the mimosis acuta, from

* *On the Mimoses*, &c.; by Marshall Hall, M.D. London, 1818.

which it may originate, or into which it may pass. The mimosis decolor is what has usually been termed *chlorosis.* Mimosis urgens, or *hysteria,* most frequently occurs as symptomatic of the mimosis decolor, or of the more continued form of the mimosis acuta. Mimosis inquieta is, in different cases, denoted by continual restlessness; wakefulness; delirium; continued rapid and hurried breathing; frequent dry cough; a sense of fluttering and hurry; some spasmodic affection; hiccough; and great frequency of the pulse. This is sometimes merely the effect of derangement in the digestive organs; sometimes it implies some obscure disease as its cause; sometimes it arises from the too copious action of a purgative, or from blood-letting; and it is occasionally the precursor of dissolution. The general causes of the mimoses are a sedentary mode of life,—particularly that of the manufacturers of lace, stockings, tambour, &c.; improper diet; watching; fatigue; anxiety; and confined and impure air. It is particularly in the manufacturers of Nottingham that Dr. Hall has witnessed the occurrence, and from whom he has taken his description of those diseases. The principal curative measures are—purgatives; appropriate diet; good air; exercise; cold-bathing, and sponging the surface of the body.

The question respecting the possibility of curing Syphilis without the use of mercury, must be considered as one of the highest importance in the practice of SURGERY. It is a question that has been frequently agitated since the period at which that disease is generally supposed to have first appeared in Europe; but it is only lately that it has led to investigations which, from the mode in which they are conducted, must (whether or not the opinion in the affirmative be well founded) be productive of the most interesting results. We have already been taught to form a more accurate diagnosis between syphilis and those diseases which, on more imperfect observation, have been considered to resemble it; we have also been taught that various constitutional symptoms,—as ulcers of the throat, eruptive disorders, nodes, and other affections of the bones,—will ensue from ulcers on the genitals contracted in coition, but which are distinct from that disease, and are curable without mercury, although they may, in their latter stages particularly, be much alleviated by that remedy; a circumstance which, from a false mode of reasoning often adopted, has added much to the obscurity in which this subject has been enveloped. And, what is of almost equal importance, it has been ascertained that syphilis is at present of such rare occurrence, that it forms a very small proportion of the number of those ulcerative diseases of the genital organs which we have daily occasion to witness.

The writers on this subject, whose observations and opinions will engage our chief attention, are Mr. CARMICHAEL and Mr. HENNEN; both gentlemen whose opportunities for observation of facts are very extensive, and whose talents are peculiarly well adapted for the investigation of this intricate subject.

Our readers, we presume, must be acquainted with the opinions which Mr. Carmichael published on this subject a short time since: in a work by that gentleman which has recently appeared,* he expresses a disposition to relinquish that one of them which is the most important in a practical point of view—that syphilis differs from other venereal diseases in not being curable without mercury. This he is disposed to do, from the powerful testimony to the contrary adduced by Mr. Guthrie, Mr. Hennen, and Mr. Rose; and also from facts which have occurred to his own observation since the period of the publication of his former work. Since then, to the date of the present one (July 1818), he has only seen three cases of syphilitic primary ulcer on the genitals: one of which occurred in a gentleman who was desirous to try whether or not it could be induced to heal without the use of mercury. Under these circumstances it remained stationary for a month, after which mercury was employed. Of the other patients, one of them came into the hospital after the primary ulcer had existed four months: it had for some time been accompanied with eruptions; the ulceration and eruptions had been extending the last month. Sarsaparilla was at this time freely exhibited: shortly after the commencement of its use, the callosity round the ulcer began to diminish and the eruptions to decline; and, at the period at which this report was published (the fifth month of the disease), the callosity was entirely removed, and the eruption so far diminished as to leave no doubt in the mind of the author of its entire disappearance, without the use of any other remedy. The other case was an ulcer of the dorsum penis, accompanied with eruptions, and pain and tenderness of the bones and joints. Sarsaparilla was given. The ulcer soon began to assume a healthy appearance in the middle, the eruptions and pains declined, and the sore, at the period at which this report was published, was "all but healed."

We are unable to compare with precision the conclusions

---

* *Observations on the Symptoms and specific Distinctions of the Venereal Diseases.*

which naturally follow from the consideration of the above observations of Mr. Carmichael, with those deduced by Mr. Hennen from his experience among the soldiers in Edinburgh castle. Since Mr. Carmichael, in his extensive practice in Dublin during so long a period, has only witnessed three cases considered by him as syphilis; and Mr. Hennen classes twenty out of 105 as cases of Hunterian chancre; it is apparently evident that those gentlemen do not agree in the characteristics which are by them considered as designative of the true syphilitic ulcer. Of the twenty cases of Hunterian chancre above referred to, nine were followed by secondary symptoms: of which, four were instances of tubercular, three of exanthematous, and one of pustular, eruptions; and one of tubercular eruptions, with ulcerated throat. As this account is taken from a tabular view of the results of the cases treated without mercury, in which particular descriptions of the symptoms are not detailed, we can only consider them as specimens of true syphilitic secondary symptoms, from such being the opinion of Mr. Hennen; but we are disposed to remark, that Mr. Carmichael has satisfactorily ascertained that *tubercular* eruptions appearing subsequently to ulcers on the genitals, do not prove the latter to have been syphilitic, although some time since so considered. This opinion is confirmed by the observations of Dr. Bateman, who has described a similar eruption arising from other diseases. This tubercular eruption, Mr. Carmichael observes, may be distinguished from syphilis, by the spots being smooth, of a small size, and rounded or much raised in the centre: whereas, the lepra syphilitica is directly the reverse; being scaly, in general of a large size, and always more raised at the circumference than at the centre.

The secondary symptoms observed by Mr. Hennen occurred more frequently, and at an earlier and more determinate period, than in cases in general in which mercury had been used; but they, in many cases, disappeared as soon; never, as has been supposed, proceeding from bad to worse, or from one succession of parts to another with unabated violence: on the contrary, they by no means exhibited the same violent and unrelenting symptoms which are observed in many instances where mercury had been employed. The eruptions did not run into ulceration, nor form into large scabs and blotches; nor were the bones of the nose or other parts, *in any instances*, affected with caries, in the many hundred cases watched by him with the utmost anxiety. These observations involve the investigation of this question in extreme perplexity. It appears so remarkable a circumstance, and one which it is so difficult to explain, how it happens that the disease, when its progress is not interfered with by the influence of mercury, should not in any instance have assumed that form so particularly insisted on by Mr. Hunter as its diagnostic character,—the regular progressive and never retrogressive course of the disease through its different stages from bad to worse, where mercury was not employed.

The non-appearance of caries of the bones, in any instance, would oblige us to attribute that affection to the influence of mercury; but such an effect is never witnessed from it when exhibited for other diseases when those parts are not affected, in equal quantities, and for a very long-continued period. The only way left to explain this is, that it is solely when disease previously existed in those parts, that mercury exercises this deleterious power. Such an opinion receives some degree of support from the diseases which ensue from the use of that mineral, when those parts are affected with scrofulous inflammation: nodes, thickening of the ligaments, ulceration of Schneider's membrane, and subsequent caries of the nasal bones, are not unfrequently observed in cases where mercury has been employed in the treatment of that disease.

The above are, we believe, the most important circumstances which the late investigations into this question have developed; a question which must by the most strenuous votaries on either part be still considered as undecided. There is, however, another point in this dispute, which we must not pass over unnoticed:—whether or not mercury possesses the power of protecting the general system against the accession of secondary symptoms? On this subject, we shall transcribe the remarks of Mr. Carmichael.

"In a question of this kind, reasoning can have no influence: we must draw our conclusions from facts, and as yet we have not a sufficient number to decide upon. In Portugal, Italy, and the southern part of Europe, Mr. Guthrie informs us, mercury is never employed for primary ulcers. My friend, Dr. Armstrong, who has resided in Vienna for many years, and is well acquainted with the practice of the most eminent physicians and surgeons of that city, informs me that they never give mercury for primary ulcers, from a knowledge that they will heal without it, and from a belief that it will not prevent the accession of constitutional symptoms.

"Mr. Guthrie's own experience is, however, very much in favour of the opposite side of the question. He informs us that the proportion of those treated without mercury, who were afterwards affected by constitutional symptoms, was under one in ten. But, according to the returns from regimental hospitals, of those treated with mercury during the half-year ending the 24th of June, 1817, not more than one in seventy-five had secondary symptoms; a statement which is greatly in favour of the exhibition of mercury, with a view to prevention. If, however, Mr. Guthrie had employed mercury in every case of true chancre only, and left all other primary ulcers to nature, it is reasonable to suppose that the proportion of cases, in which secondary symptoms occurred, would be very considerably lessened."*

The results of the mode of treatment adopted at the hospital at Stockholm, by Mr. OSBECK,† do not furnish us with any useful facts respecting this subject; since, according to the report of Prof. SCHWEIGGER,‡ Mr. Osbeck selected his cases from those of long standing; and in many of them mercury had been employed unsuccessfully: they were, indeed, specimens of that disease which is very common in Norway and along the coasts of Sweden, termed *radefyge* by the Danes, and *saltfluss* by the Swedes; and which Prof. Schweigger considers to be the same with the *sibbens* of Scotland. This disease, he observes, is met with in several parts of Prussia, without being distinguished by any particular name, and is still common in Courland and Livonia: it is generally considered by the vulgar as a degenerated species of syphilis. This opinion of Prof. Schweigger accords with that expressed by Dr. Hamilton in an interesting Memoir published by him on this subject.§

Dr. Hamilton, however, carries this opinion still further; considering that the disease which first appeared in Europe at the latter part of the fifteenth century, was also of this nature, and not the *lues* of Hunter; and that, therefore, the writings of the greater number of the surgeons of that and the immediately subsequent period are not applicable to true syphilis.

Prof. Schweigger visited the hospital at Stockholm, which was intended for sixty patients, but only twelve were in it at that time, (probably from its being the season of the harvest.) One of them was a boy *twelve years* of age, and who had been three years affected with the disease for the cure of which he was admitted, which was evidently radefyge.

* *Op. citat.* p. 25.

† *London Medical and Physical Journal*, vol. xl. p. 349.

‡ *Journal der Praktischen Heilkunde.*

§ *Edinburgh Medical and Surgical Journal*, vol. lv.; and *London Medical and Physical Journal*, vol. xl. p. 546.

We have already mentioned the opinion of Mr. Carmichael,—that syphilis is now comparatively seldom to be witnessed in this country; although he thinks there is reason to believe that it was the most predominant form of the venereal disease in the time of Hunter. He also remarks, that "a few years back, and particularly in 1812 and 1813, that malignant attendant of the phagedenic disease, the sloughing ulcer, was extremely prevalent in Dublin; but I have not seen it in a single case, either in private or public practice, during the last two years. It may, therefore, be considered as far from improbable that there are prevailing forms of venereal affections, like prevailing epidemics, at different times and in different countries; a circumstance that may possibly depend on the importation of fresh infections, although these arrivals are seldom recorded with the same notoriety as that of syphilis from St. Domingo."*

This opinion of Mr. Carmichael corresponds with that of Dr. Weizmann, a physician of eminent talents, residing at Bucharest.† Dr. W. also considers that infectious diseases of this kind occasionally appear, of sporadic origin, from the influence of strange climates, and connexion with persons of a different nation. Extensive series of observations, made in Moldavia, Wallachia, and Bulgaria, have led him to believe that ulcers on the genitals arising from common causes, frequently appear; which would heal readily by simple means, if attention were paid to them; but which, if venereal intercourse be indulged in, degenerate into chancres, are followed by pains of the bones, nodes, and indeed all the ordinary symptoms of true syphilis; and in many instances cannot, apparently, be cured without mercury, although the patients have only had connexion with women, not only (as far as could be discovered) free from disease, but in whom it could not be supposed possible that a disease of that nature could exist. These cases were witnessed in countries where women are carefully confined in harems, and affected men who only had venereal intercourse with women thus secluded.

Dr. Weizmann mentions having been consulted by several persons of distinction at different times, who, on the night of their nuptials had contracted blenorrhagia, chancres, and buboes, from their wives; of whose virginity there could not exist a doubt, but who, perhaps, were affected with leucorrhœa. He relates the case of a Pacha who was

---

* *Op. citat.* p. 220.

† *Russiche Sammlung für Naturwissenschaft und Heilkunde,* tome i. p. 126.

affected with the ordinary symptoms of syphilis, as well as his twenty-four wives, although he had not had venereal intercourse with any excepting them, but several of whom had for some time previously had leucorrhœa; and the pacha himself was afflicted every summer with an eruption, which first appeared on the genitals, and afterwards over the whole of the body. This Dr. W. attributes to his not having abstained from coition during that season of the year.

This author also endeavours to show that infectious diseases of this kind have been prevalent in those countries for two thousand years; and he observes, that many of the physicians believe that syphilis has existed there for so long a period; and that, in the second century of the common æra, when Trajan sent a number of colonies into Dacia, many of the persons constituting them became affected with an ulcerative disease on the genitals, which spread over the country with great rapidity; and, when permitted to proceed without interruption, frequently terminated in death.

Since the labours of Faber, Duverney, Petit, Desault, Boyer, Pott, Kirkland, White, Callisen, and Richerand, but little is left to be desired respecting the history of dislocation of the bones of the extremities: some varieties of these accidents have, however, been recorded by systematic writers, rather as possible than as what has actually occurred; amongst which is the complete dislocation of the tibia anteriorly from the femur. Several authors have considered it as not of possible occurrence; others have supposed that, were it to happen, amputation would be decidedly indicated. The following account of an instance of the species of injury above alluded to, and the favourable mode in which it terminated, must be considered particularly interesting. It is related by M. J. Lavalette.* The accident arose from the falling of a mound of earth on the subject of it, while at work in preparing fortifications. It was accompanied with a turning inwards of the foot; the limb was nearly extended, and somewhat shortened; and there was a considerable depression above the condyles of the femur: the patella was turned upward and horizontally, so that its inferior margin was placed anteriorly, and it lay on the surface of the head of the tibia; and there was fracture of the fibula near its upper extremity. Flexion and extension of the limb could be effected, without being productive of very

* *Journal complémentaire du Dictionaire des Sciences Medicales,* tome 1.

great pain. It was reduced without much difficulty, and at the end of six weeks the patient had regained the perfect power of motion of the joint; but some degree of weakness of the flexor muscles of the foot remained; and, in walking, the limb was moved in a rotatory manner, describing nearly a circle, which appeared to be owing to rupture of the transverse ligaments of the joints. The power of flexion and extension of the leg on the thigh, observed in this case, will perhaps afford the pathognomonic sign between this accident and incomplete dislocation.

Separation of the epiphyses of the long bones is generally considered as of rare occurrence, and possible only in very early age. Boyer considered that cases stated to be of this kind were always instances of fracture of the neck of the bone. M. Laurent has made public an account of two accidents of this sort, that occurred to the observation of Dr. Champion of Bar-le-Duc.* One happened in a boy eleven years of age, who, falling from a chariot, had his arm caught in the wheel, and was drawn by it for some distance. The skin was separated from the greater part of the arm; but the muscles and other soft parts were not apparently injured. Fracture of the bone was not suspected. It was on the third day from the accident when Dr. Champion saw the patient: a considerable degree of inflammation had then taken place; on the seventh day he died. The epiphysis of the scapular extremity of the humerus was found to be separated from the body of that bone, but remained in its natural situation with respect to the joint. Some degree of laceration of the capsular ligament and synovial membrane had taken place.

The other case occurred in a boy thirteen years of age: it arose from his fore-arm having been caught in the card of a cotton machine, by which it was twisted so as to have performed almost entire rotation with respect to the arm, just above the elbow-joint. The soft parts were so much injured that amputation was considered necessary. The cubito-radial epyphysis was found to be entirely separated from the os humeri, having a small flake of that bone attached to it. The epiphysis preserved its natural situation with respect to the fore-arm, being thus retained by means of the lateral ligaments: the anterior and posterior ligaments and the synovial membrane were torn off at the place of the disjunction of the epiphysis.

---

* *Journal complémentaire du Dict. des Sciences Medicales*, tome 1.

It has generally been considered that when hydrocele of the tunica vaginalis testis became cured after the evacuation of the fluid by simple puncture, that it arose from the cavity becoming obliterated; and Mr. Elsa on observing that simple puncture succeeded more frequently formerly than at present, remarked that this arose from the larger size of the puncture then made producing sufficient inflammation to cause adhesion of the tunics. Mr. Kinder Wood, from whom we take these observations, remarks,* that Mr. Pott pointed out the true cause of this result,—when he observes that, if the incision healed immediately, the disease would return; but, if it became inflamed and sloughy, adhesion of the tunica vaginalis to the albuginea took place. Some cases lately witnessed by Mr. Wood have enabled him to ascertain that adhesion between those tunics does not happen in all cases cured in the above manner; but that, on a certain degree of inflammation being excited in the sac, the exhalant vessels are roused to more vigorous and healthy action,—thus overcoming that relaxed and atonic state of them which is the true cause of the accumulation. The fact of the absorption of blood taking place from the sac of an hydrocele, which is evident in the twenty-fifth case related by Sir James Earle, and yet the accumulation of fluid at the same time going on, shows that the disease cannot be explained by the debility of the absorbents.

To stimulate the parts, or induce a slight degree of inflammation, Mr. Wood continues to observe, would appear to be the most obvious means of cure; and this may be effected by preventing the puncture from healing by the first intention; in which case, as in openings into the cavities of the pleura, peritoneum, joints, &c. the inflammation is found to extend from the wound over the membrane lining the cavity. Some cases were successfully treated by Mr. Wood from those indications, by making the incision with a lancet, and afterwards drawing out and excising a portion of the tunica vaginalis.

Since the publication of Mr. Brodie's new mode of dividing varicose veins, several cases have been treated in this manner by Mr. Carmichael, of Dublin,† with complete success. He gives a detail of six, and mentions that he has employed this practice in several other instances, which he

---

* *Medico-Chirurgical Transactions,* vol. ix. part 1.

† *Transactions of the Association of the Fellows and Licentiates of the College of Physicians in Dublin,* vol. ii.

does not relate, because he did not take notes respecting them. In five of the cases, the veins were divided just below the knee: in one, the trunk of the saphena was operated on with Mr. Brodie's knife, and according to his plan. In this instance, considerable symptomatic fever, with great restlessness and total want of sleep, hard and quick pulse, and brown and furred tongue, appeared on the sixth day from the operation. These symptoms were relieved by bloodletting, and the application of bread-and-water poultices.

These cases are a useful addition to those related by Mr. Brodie, and confirm the superior advantages attending his mode of operating; they also powerfully support his opinion, that equal danger does not attend a wound of the branches as an injury of the trunk of the vein.

We have already noticed the work of Mr. Brodie on Diseases of the Joints, and the same reasons we before gave for not entering into the consideration of it in a pathological point of view will apply to it on the present occasion. We feel, however, disposed to remark, that it strongly evinces the advantages that are to be derived from the study of pathological anatomy. Although no new particular remedies nor general modes of treatment, are proposed, yet it must be considered of the highest value in the practice of surgery. This arises from the precise views it gives of the diseases of which it treats; of their origin and progress; and of the particular measures that are applicable to particular forms of disease.

The beneficial powers of the most efficacious remedy with which we are acquainted, for some diseases of the joints, were known to the earliest writer on Surgery, and it has been employed nearly down to the present time with as little precision, in general, as that which directed him in the use of it: it has, necessarily, been productive of much benefit; but, like all other powerful measures not judiciously employed, it has caused almost equal mischief. The German surgeons have studied the diseases of the joints with considerable assiduity, and we cannot readily forget the lecture of Prof. Schreger on opening his Clinical School at Erlangen: but, in the latest and best of their works on this subject,* we do not find that precision we are taught by

---

* *Arthrokakologie, oder über die verrenkungen durch innere bedingung, und über die Heilkraft wirkungs-und anwendungsart des Glüheisens bey diesen Krankheitsformen.* Von Johann Nepomuk Rust.—We are preparing an analysis of this work for early insertion in the Foreign Department of our Journal.

Mr. Brodie for the application of the various remedies appropriate to the different forms and stages of the diseases of those organs, from the want of that pathological knowledge which he has acquired.

A mode of treating spasmodic constriction of the anus, that has been attended with remarkable success, has lately been adopted by M. Boyer.* Some of the cases in which he employed it were accompanied with a peculiar fissure or groove, the surface of which was excoriated, running longitudinally up the intestine. This fissure, M. Boyer observes, appears not to have been previously observed with attention, since it is not accurately described by any former writer. Albucasis, Sabatier, and Lemonnier, make mention of fissures and rhagades of the anus; but, from the means recommended for their relief by those authors, which consisted principally of unguents, being stated by them to have cured the disease, M. Boyer considers that it could not have been similar to that of which he treats; since the patients who came under his care with this malady had employed every variety of application that could be suggested to be capable of procuring relief, without thence receiving any benefit. Adults appear to be exclusively subject to this disease; the greater number of patients affected with it were from twenty-five to forty years of age. No class of society is exempt from it, and women seem to be somewhat more frequently affected than men. The characteristic sign of the disease is a fixed pain in some point surrounding the anus, which is always increased during the evacuation of the fæces; which is effected with extreme difficulty. The sphincter of the anus is so contracted, that the introduction of a finger or a bougie is very difficult, and productive of excessive pain. The causes of this affection are very obscure: in many cases it has been preceded by hæmorrhoidal tumefaction, and in some instances excision of such tumors had been previously performed. Nothing remarkable can be seen externally: in some cases, hæmorrhoidal tumors, or small protrusions, have been witnessed, but they were not considered by M. Boyer to be connected with the fissure. In some instances, the extremity of the fissure may be perceived at the orifice of the anus; but most frequently it can only be discovered by separating the sides of the rectum for some distance upwards by the fingers.

---

* *Journal complémentaire du Dictionaire des Sciences Medicales*, tome ii.

When the finger is introduced, a powerful constriction on it is felt; and the patient experiences extreme pain on pressure on some particular part, which is that of the situation of the fissure. M. Boyer has some doubts respecting which of those two affections should be considered as the original disease, and is inclined to believe that the fissure is a consequence of the spasmodic constriction; since the former never exists without the latter, whilst this frequently occurs without the fissure. No medicinal applications were found to be productive of benefit; but the disease was, in every instance, removed by the division of the contracted part of the intestine with a bistoury. This division was always effected at one or other of the sides of the anus, where the fissure is usually situated, and not anteriorly or posteriorly: should the fissure, however, occur in either of the latter situations, it must not alter the mode of performing the operation. On the third day previous to the operation, a gentle purgative is to be administered, and on the same day a glyster; in order that the patient may remain for three or four days without any fæcal evacuation. He is to lay on his side, in the same manner as for the operation for fistula: the forefinger is then to be oiled, and introduced into the rectum; a narrow bistoury, rounded at the extremity, is to be passed along the finger, with one of its broad surfaces laying flat on it; the cutting edge is then to be turned towards the surface of the intestine, in the course of the fissure, if it be situated laterally; and the coats of the intestine, sphincter muscles, and the surrounding cellular tissue, divided at one stroke. A triangular wound is thus made, the summit of which corresponds with the intestine, and the base with the skin: it may, perhaps, be necessary to extend this part of it, which may be done with another stroke of the instrument. In some cases, the intestine slides before the edge of the bistoury, and the wound of the cellular structure is then longer than that of the intestine: it will be necessary here to introduce the bistoury again, to lengthen the wound of the intestine; or to effect this with blunt-pointed scissars.

When the constriction is very great, two incisions, one on each side, should be made. A large bougie is to be introduced into the parts, to prevent irregular adhesion of the wound; the external dressing is of the usual kind for similar operations. The wound is generally cicatrized in about a month or six weeks; in a few instances it has not been effected until between the second and third month. All the patients thus operated on, fifty in number, were perfectly and permanently cured.

The sixth volume of the Treatise on Surgical Diseases of M. le Baron Boyer has been lately published, which nearly completes a work that will ever be referred to as the standard of the state of surgery at the present period. We do not find in it any striking novelties in theory or modes of practice; nor of those attempts, so frequently witnessed, to spring without the limits of our knowledge, without the ability to extend its boundaries; but, information which is of real utility, and calculated to improve the practice of surgery. It contains descriptions of diseases, which will render them intelligible to the practitioner when he is called to witness them at the bed-side of the patient; and directions for his conduct, that will not be found mere illusions when they become the subject of experiment. The nature of our plan will not permit us to enter into the particular consideration of this work on the present occasion; but we shall, at a future period, furnish our readers with an analysis of the most valuable part of its contents.

The art of forming "supplemental noses," that has lately been revived in Europe, must be regarded as an object of considerable interest in the practice of surgery. The absence of the intellectual character of the human countenance which accompanies the want of that feature; and, what is of greater consequence, the uncleanliness attending it, and the disagreeable ideas respecting its origin that are associated with its appearance, render the removal of that deformity a circumstance of much importance to persons obliged to communicate with civil society. Mr. Carpue, who brought this practice again into the repute it has acquired in England, has adopted the *Indian method;* but that employed by Tagliacozzi, with some modifications, is preferred by Professor Graefe, of Berlin, after trials of both of them in several instances.* Prof. Graefe first had recourse to this practice in the year 1811, when he employed the Indian mode; again in 1816, according to the Italian; in 1817, after the Indian; and again in the same year, in the manner we have mentioned as that preferred by him, and which he is disposed to term the *German method.* The

---

* *De Rhinoplastice sive Arte curtum Nasum ad Vivum restituendi, commentatio, qua prisca illius ratio iterum experimentis illustratur, novisque methodis ad majorem perfectionem perducitur.* Conscripsit Carolus Ferdinandus Græfe; Latine edidit J. F. C. Hecker. *Berolini,* apud Reimerum; et *Londini,* apud Treuttel et Würtz. pp. 161. 1818.

last instance determined his choice; and, making some allowance for warmth of expression in describing a favourite measure, it would thence appear that the latter method is entitled to preference. The patient was a female, twenty-four years of age, who had the cartilaginous portion of the nose destroyed by herpes. "The operation," he observes, "succeeded so completely in every respect, that no person could observe the least deformity: the new parts perfectly assimilating to the general agreeable appearance of the young lady's countenance." Such a description would be by no means appropriate to the results of the operation in the instances in which it has been performed in England. Other advantages, the author observes, attend this method; since the cicatrix on the forehead, and danger of ill consequences from exposure of the frontal bone, are thus avoided.

We have to notice the progress of a work that has contributed in an eminent degree to the improvement of medicine, and which will, when completed, constitute a record of the degree of perfection to which that science has arrived at the existing period; and then our labours on the present occasion terminate. The *Dictionaire des Sciences Médicales* will, doubtless, long maintain this character, and will convey to future ages a testimony of the talents of its authors, honourable to the distinguished nation that gave them birth, and highly gratifying to those engaged in the cultivation of the branch of science most interesting to human nature.

H.

*Sackville-street; December* 31, 1818.

### CORRIGENDA.

Page 5, line 29, after *Cloquet* add *Hippolytus.*

— 12, — 22, for *stratum* read *striatum.*

— 19, — 9, after *facts* add *these.*

— 24, — 24, after *action* add *solely.*

— 26, — last line, for *action* read *actions.*

